# In Situ Growth of Robust 2D ZIF‐67 MOF in Block Copolymer Membranes for Ultrafast Molecular Degradation

**DOI:** 10.1002/advs.202416169

**Published:** 2025-02-18

**Authors:** Jingjing Xu, Jianyong Yu, Leiming Guo, Faxue Li, Nikos Hadjichristidis

**Affiliations:** ^1^ Key Laboratory of Textile Science & Technology Ministry of Education College of Textiles Donghua University Shanghai 201620 China; ^2^ Innovation Center for Textile Science and Technology Donghua University Shanghai 201620 China; ^3^ Polymer Synthesis Laboratory KAUST Catalysis Center Physical Science and Engineering Division King Abdullah University of Science and Technology (KAUST) Thuwal 23955 Saudi Arabia

**Keywords:** 2D MOF, advanced oxidation process, block copolymer, membrane reactor, molecular degradation

## Abstract

Membrane‐based advanced oxidation processes (AOPs) rely heavily on the configuration of membrane structures and catalysts. However, designing state‐of‐the‐art membrane structures integrated with tailored catalysts for efficient AOPs remains a significant challenge. In this study, for the first time, hybrid membranes are constructed by the in situ growth of 2D ZIF‐67 onto the nanopore walls of 3D block copolymer (BCP) membranes. These membranes feature highly tunable pore structures, leading to exceptional catalytic performance that surpasses previously reported membranes. The remarkable catalytic efficiency stems from the predominant role of the non‐radical species, ^1^O_2_, in catalytic degradation, combined with the integration of the high‐surface‐area 2D ZIF‐67 and the tortuous pore structures of the BCP membranes. The resulting catalytic membranes demonstrate robust performance, achieving stable permeance of over 1800 L (m^2^·bar·h)^−1^ while completely degrading dyes during long‐term filtration. Notably, the degradation efficiency is maintained at 90% even when the permeance is adjusted to 3070 L (m^2^·bar·h)^−1^. Additionally, the membranes exhibit excellent resistance to both alkali and acidic environments and are unaffected by various background anions or the types of degraded molecules. This work presents a novel approach to designing advanced catalytic membranes for high‐efficiency, space‐confined AOPs.

## Graphical abstract

The robust membranes with highly adjustable catalytic performance are prepared for the first time by the in situ growth of 2D ZIF‐67 MOF onto the nanopore walls of 3D block copolymer (BCP) membranes. These membranes can continuously degrade 100% of small molecules while maintaining a permeance exceeding 1800 L (m^2^ **·**bar**·**h)^−1^, demonstrating exceptional efficiency and durability for water treatment applications.

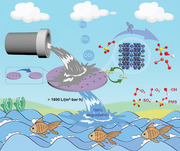

## 1 Introduction

Water scarcity is a serious threat to the survival of humans, animals, and plants on our planet.^[^ ^1^ ^]^ Moreover, water shortages caused by water pollution have become a prominent issue in recent decades due to rapid industrial development.^[^ ^2^ ^]^ Industrial effluents often contain various pollutants; however, particular attention must be paid to small molecules, such as synthetic dyes discharged by dyeing industries. These small molecules tend to migrate from water into human food chains, significantly increasing the risk of cancer in humans.^[^ ^2^, ^3^ ^]^ Additionally, these pollutants are highly toxic to animals and plants, and their effects often lead to the death of aquatic life, thereby exacerbating water pollution and severely endangering ecological balance.^[^ ^4^ ^]^ Therefore, an efficient method is urgently needed to eliminate small molecules from polluted water and mitigate their harmful impacts.

AOPs are widely accepted as an important and highly efficient strategy for wastewater treatment.^[^ ^5^ ^]^ Unlike technologies such as separation and adsorption, AOPs have the unique ability to convert pollutants in wastewater into harmless products like CO_2_ and H_2_O rather than merely removing them.^[^ ^6^ ^]^ The free and/or non‐free radicals generated by AOPs exhibit strong oxidative capabilities, making them effective for degrading recalcitrant pollutants.^[^ ^7^ ^]^ These radicals, commonly referred to as reactive oxygen species (ROS), face a significant limitation due to their short lifetime. For example, the hydroxyl radicals (**·**OH) produced by traditional Fenton reactions have a lifespan of less than 10 µs.^[^ ^8^ ^]^ Such extremely short lifetime hinder the efficiency of AOPs, as most ROS become inactivated before they can interact with pollutants in water. To address this challenge and reduce the ineffective consumption of ROS, membrane technology has been integrated with AOPs to construct heterogeneous systems by anchoring catalysts onto membrane pore walls.^[^ ^9^ ^]^ This approach confines pollutant degradation within the membrane pores as wastewater flows through, facilitating continuous degradation. Moreover, the degradation efficiency is significantly enhanced due to space‐confined catalysis at the nanoscale or even angstrom scale.^[^ ^10^ ^]^

The integration of porous membranes with catalysts for space‐confined AOPs necessitates the careful design of membrane pore structures. The pore size of membranes determines the catalytic reaction space and significantly influences degradation efficiency.^[^ ^8^ ^]^ Narrower pores, in principle, reduce the distance between the catalysts anchored on the membrane pore walls and the pollutants carried by water. Consequently, ROS derived from catalyst‐activated oxidants (e.g., H_2_O_2_) have a higher likelihood of oxidizing pollutants before becoming inactivated in water. For instance, the typical anodic alumina oxide (AAO) membranes^[^ ^8^ ^]^ and ZrO_2_/TiO_2_ ceramic membranes,^[^ ^11^ ^]^ commonly employed as high‐efficiency reactors for AOPs, are often characterized by long (tens of µm) and narrow pores (≈20 nm or smaller in size). These structural features enable rapid pollutant oxidation via ·OH radicals generated within the membrane pores. To meet the growing demand for simultaneous enhancement of permeance and degradation efficiency, state‐of‐the‐art 2D material membranes with fast water transport and narrow mass transfer channels have been proposed.^[^ ^10^, ^12^ ^]^ 2D membranes constructed from catalyst‐contained 2D nanosheets offer superior performance in AOPs, particularly when utilizing persulfate salts as oxidants instead of traditional H_2_O_2_.^[^ ^13^ ^]^ However, while the extremely narrow channels of 2D membrane reactors improve degradation efficiency, they inevitably limit permeance to relatively low levels.^[^ ^14^ ^]^ Thus, the challenge of designing advanced membrane structures decorated with optimized catalysts for fast and efficient AOPs remains an area requiring further exploration and innovation.

MOFs represent a unique class of porous materials constructed by the coordination of metal ions and surrounding organic linkers.^[^ ^15^ ^]^ Metal catalyst‐containing MOFs have been intensively used in AOPs in recent years owing to their intrinsic porous nature, high surface area, and abundant active sites.^[^ ^6^, ^16^ ^]^ Among them, 2D MOFs are particularly advantageous, as their more accessible active sites facilitate efficient molecular diffusion,^[^ ^17^ ^]^ leading to enhanced catalytic performance.^[^ ^18^ ^]^ However, preparing defect‐free 2D MOF membranes for AOPs remains highly challenging due to the limited hydrolytic stability of MOFs.^[^ ^19^ ^]^ An alternative approach involves the in situ growth of 2D MOF catalysts on the pore walls of highly permeable matrix membranes.

On the other hand, BCPs, consisting of two or more physiochemically distinct homopolymer blocks, are increasingly recognized as promising next‐generation membrane materials.^[^ ^20^ ^]^ Their adaptability to various pore‐forming strategies allows the creation of porous structures with highly tunable pore sizes^[^ ^21^ ^]^ while amphiphilic BCPs are easily processed into highly permeable membranes.^[^ ^22^ ^]^ The hydrophilic chains enriched onto pore walls of BCP membranes have been demonstrated to readily coordinate with metal ions,^[^ ^23^ ^]^ offering a significant opportunity for the in situ growth of metal‐containing 2D MOFs. Membrane reactors composed of 2D MOF catalysts anchored to the pore walls of 3D BCP membranes for AOPs hold immense potential for achieving remarkable pollutant degradation efficiency and enhanced permeability. Such catalytic membranes with highly tunable pore sizes offer a promising way to break though the trade‐off between degradation efficiency and permeability remained in the conventional catalytic membranes. However, to the best of our knowledge, this innovative concept has not yet been reported.

As a proof of concept, this work demonstrates the in situ synthesis of 2D Co‐containing MOFs, specifically ZIF‐67, onto the nanopore walls of BCP membranes composed of polystyrene‐*b*‐poly(4‐vinylpyridine) (PS‐*b*‐P4VP) via hydrothermal synthesis. The resulting membrane reactors for AOPs exhibit performance that significantly outperforms previously reported catalytic membranes. The BCP membranes were fabricated using the “retarded dissolution” pore‐forming strategy developed in our earlier work.^[^ ^22a^ ^]^ Without undergoing drying, the membranes, freshly taken from methanol, were directly immersed in an aqueous solution of ZIF‐67 precursors for in situ synthesis of 2D ZIF‐67. The resulting BCP/ZIF‐67 hybrid membranes, with a pore size of 70 nm, were employed in peroxymonosulfate (PMS)‐based AOPs. These membranes demonstrated an impressive permeance of 1866 L (m^2^·bar·h)^−1^ while achieving 100% degradation of dyes.

The performance of the catalytic membranes can be easily adjusted by modulating the pore‐forming process of the BCP membranes. For instance, even at permeances as high as 3070 and 3800 L (m^2^ **·**bar**·**h)^−1^, the degradation efficiencies of dyes remain 90% and 83%, respectively. Furthermore, the catalytic membranes exhibited excellent robustness, withstanding a wide pH range of 4 to 10, and demonstrated long‐term stability with minimal Co leaching (2.35 µg L^−1^) after ≈70 h of filtration. Subsequently, the degradation applicability for other small molecules and insights into the degradation mechanism were explored. This work challenges the conventional notion that smaller membrane pores correlate with higher catalytic efficiency, offering a new pathway for the synthesis of advanced catalytic membranes for the rapid and efficient purification of wastewater.

## 2 Results and Discussion

**Figure 1:**
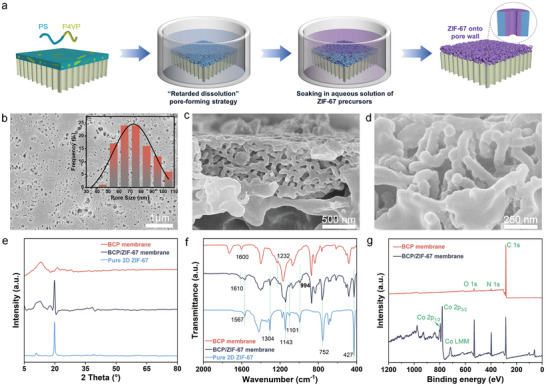
The preparation and structural characterizations of the BCP/ZIF‐67 membranes. a) Schematic illustration of the fabrication process for the BCP/ZIF‐67 hybrid membranes. b) SEM surface and c) cross‐section images of the BCP/ZIF‐67 membranes. d) Enlarged view of the cross‐section of the BCP/ZIF‐67 membranes. e–g) XRD patterns (e), FT‐IR spectra (f), and XPS wide‐scan spectra (g) of the BCP/ZIF‐67 membranes. The inset of (b) is the pore size distribution of the BCP/ZIF‐67 membranes.

The sphere‐forming PS‐*b*‐P4VP (*M*_n_^PS^ = 120 kg mol^−1^, *M*_n_^P4VP^ = 20 kg mol^−1^) was dissolved in chloroform to prepare 2 wt% solutions, which were then spin‐coated onto hydrophilic polyvinylidene fluoride (PVDF) substrates with an average pore diameter of 0.22 µm.^[^ ^22a^ ^]^ The resulting composites have solid BCP top layers with an initial thickness of ≈257 nm, as determined using a film thickness gauge. The composites were then immersed into the mixed solvent containing toluene and methanol for varying durations, followed by quenching in methanol for arbitrary periods.^[^ ^22a^ ^]^ Without undergoing a drying process, the methanol‐quenched composites were immediately soaked into the aqueous solution of 2D ZIF‐67 precursors,^[^ ^24^ ^]^ the mixtures of Co(NO_3_)_2_·6H_2_O and 2‐methylimidazole, for 1 h (**Figure** **1**a). This approach allowed the ZIF‐67 precursors to coordinate with the stretched P4VP chains, facilitating the in situ growth of 2D ZIF‐67 onto the nanopore walls of the PS‐*b*‐P4VP membranes. The resulting BCP/ZIF‐67 hybrid membranes were subsequently fabricated after a drying step. Notably, the selection of 2D ZIF‐67 as the catalyst lies in its strong chemical stability, high catalytic activity as well as large specific surface area.^[^ ^24^ ^]^ Moreover, the highly dispersed Co (II) active sites in ZIF‐67 can effectively mitigate Co ion leaching.^[^ ^25^ ^]^ And the remarkable capability to activate PMS by ZIF‐67 has been further demonstrated.^[^ ^26^ ^]^

Additionally, the highly porous interconnected nanopores of the BCP membranes were generated using the “retarded dissolution” pore‐forming strategy in our previous work, which required complete drying of the methanol‐quenched membranes before pore formation.^[^ ^22a^ ^]^ However, dried BCP proved unsuitable as substrates for the growth of 2D ZIF‐67 (Figure S1, Supporting Information). We hypothesized that the highly stretched P4VP chains of the BCP, aligned along methanol pathways during methanol quenching, favor subsequent coordination of the 2D ZIF‐67 precursors in aqueous solutions. Therefore, methanol‐quenched BCP membranes, without undergoing drying, were selected as substrates for the in situ growth of 2D ZIF‐67 in this study.

Here, the composites derived from spin‐coating BCP onto PVDF substrates were treated in a toluene/methanol mixture (v/v = 35/65) for 3 s, followed by quenching in methanol. Subsequent soaking in aqueous solutions of 2D ZIF‐67 precursors for 1 h resulted in the formation of BCP/ZIF‐67 membranes enriched with numerous round‐like nanopores on the surface (Figure 1b). The average pore size of these hybrid membranes was determined to be ≈70 nm, significantly smaller than that of the BCP membranes without growth of ZIF‐67 (≈80 nm) (Figure S1a, Supporting Information). Moreover, the surface porosity of the BCP/ZIF‐67 membranes was merely 7.7%, much lower than that of the BCP membranes (≈30.5%) (Figure S2, Supporting Information). This reduction in pore size and surface porosity highlights the successful growth of ZIF‐67 within the BCP membranes. Cross‐sectional analysis using scanning electron microscopy (SEM) provided further insights into the hybrid membranes (Figure 1c,d). The pore skeletons of the hybrid membranes appeared significantly smoother and greatly enlarged compared to those of the pristine BCP membranes (Figure S3, Supporting Information), indicating the homogeneous growth of 2D ZIF‐67 in the BCP membranes. The thickness of the BCP/ZIF‐67 membranes was determined to be ≈828 nm, slightly less than the BCP membranes (≈970 nm). This difference can be attributed to the quenching solvent, methanol, which exhibits a stronger affinity for P4VP than water. As a result, the stretched polymer chains of PS‐*b*‐P4VP, initially soaked in the toluene/methanol mixture, tend to shrink more in the aqueous solutions of ZIF‐67 precursors than in pure methanol. Consequently, the membranes became thinner despite the growth of 2D ZIF‐67 on the pore walls. To further illustrate the growth of 2D ZIF‐67 within the BCP membranes, the soaking time was extended to 12 h, resulting in the formation of thicker and larger 2D MOF sheets (Figure S4, Supporting Information).^[^ ^24^ ^]^

The mechanical stability of the BCP/ZIF‐67 membranes was subsequently evaluated. Compared to the BCP membrane and the PVDF support, the tensile strength of the BCP/ZIF‐67 membrane was greatly enhanced (Figure S5, Supporting Information). This is ascribed to the conformal growth of the thin 2D ZIF‐67 layers onto the BCP membrane pore walls though in situ synthesis. Meanwhile, the flexibility of the membranes was not deteriorated after growth of ZIF‐67, implying the superior strategy of in situ synthesis for preparation of the BCP/ZIF‐67 membranes. The high mechanical stability of the hybrid membrane is undoubtedly in favor of membrane‐based AOPs.

To further confirm the growth of 2D ZIF‐67 within the BCP membranes, X‐ray diffraction (XRD) was used to compare the crystal structure of the BCP membranes with that of the BCP/ZIF‐67 membranes (Figure 1e). The pure 2D ZIF‐67 exhibited peaks at 10.8°, 19.7°, and 39°, consistent with previous works.^[^ ^27^ ^]^ The diffraction peak at 19.7° appeared clearly in the XRD pattern of the BCP/ZIF‐67 membranes but was absent in the BCP membranes, providing direct evidence of 2D ZIF‐67 growth on the nanopore walls of the BCP membranes.^[^ ^27a^ ^]^

The chemical structures of the BCP/ZIF‐67 membranes were further analyzed using Fourier transform infrared spectroscopy (FTIR) (Figure 1f). The peak centered at 1232 cm^−1^ corresponds to the C‐H deformation of the P4VP block.^[^ ^28^ ^]^ The characteristic ring‐stretching mode of pyridine (P4VP) was located at 1600 cm^−1^, which shifted to a higher wavenumber at 1610 cm^−1^ after 2D ZIF‐67 growth, indicating coordination between Co^2+^ and pyridine rings of P4VP.^[^ ^29^ ^]^ Moreover, the formation of Co‐N bonds during the synthesis of 2D ZIF‐67 resulted in a new peak at 994 cm^−1^ in the spectrum of the BCP/ZIF‐67 membranes. A strong peak at 427 cm^−1^, associated with Co‐N vibration bonds,^[^ ^30^ ^]^ was present in the spectra of both the BCP/ZIF‐67 membranes and pure 2D ZIF‐67, further confirming the synthesis of 2D ZIF‐67 within the BCP membranes. The formation of 2D ZIF‐67 in the BCP membranes was further supported by the presence of the characteristic in‐plane bending centered at 1101 cm^−1^ peak, which originates exclusively from the free 2‐methylimidazolate of 2D ZIF‐67.^[^ ^31^ ^]^ Additionally, in the spectrum of the BCP/ZIF‐67 membranes, a peak at 1567 cm^−1^ was observed corresponding to the stretching vibrations of C = N in the 2‐methylimidazolate ligand.^[^ ^32^ ^]^ A series of peaks ranging from 600 to 1500 cm^−1^ (752, 1143, 1304 cm^−1^) were also identified, which correspond to the out‐of‐plane vibration of the imidazole ring.^[^ ^33^ ^]^ These findings, together with the XRD patterns, confirm the successful in situ growth of 2D ZIF‐67 within the BCP membranes.

The surface composition of the synthesized BCP/ZIF‐67 membranes was then monitored by X‐ray photoelectron spectroscopy (XPS) (Figure 1g). A comparison of the wide‐scan XPS spectra of the BCP membranes, with and without ZIF‐67, revealed the presence of Co 2p_3/2_ (781.4 eV) and Co 2p_1/2_ (797.2 eV) orbital doublet peaks exclusively in the BCP/ZIF‐67 membranes. These two peaks, attributed to Co^2+^,^[^ ^34^ ^]^ further corroborate the in situ formation of 2D ZIF‐67 within the membranes. The homogeneous distribution of Co across the membrane cross‐section provides additional evidence of uniform growth of 2D ZIF‐67 within the BCP membranes (Figure S6, Supporting Information).

**Figure 2:**
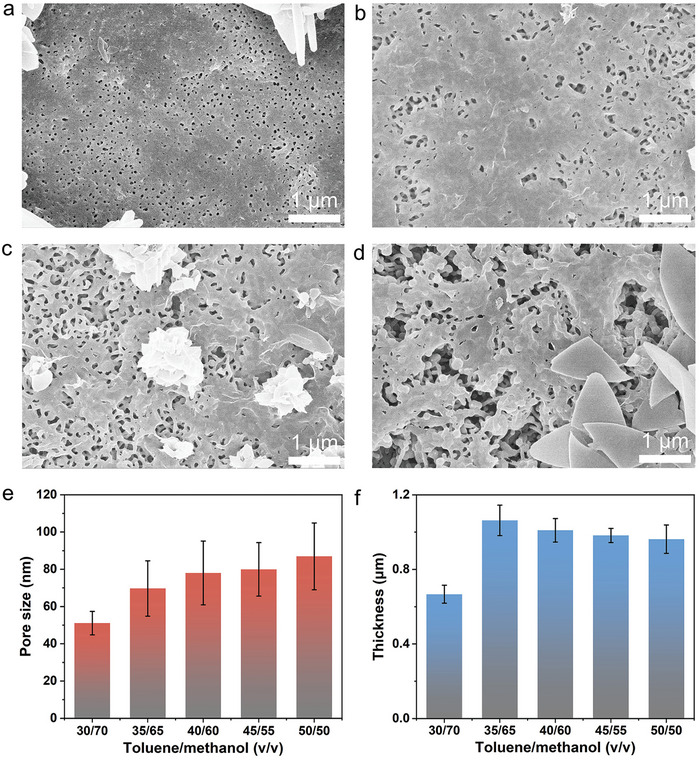
The morphology and the structural parameters of the BCP/ZIF‐67 membranes. SEM surface images of the BCP/ZIF‐67 membranes soaked in mixed solvents containing toluene and methanol with the volume ratios of a) 30/70, b) 40/60, c) 45/55, and d) 50/50 for 3 s, respectively. Histograms of pore size (e) and thickness (f) of the BCP/ZIF‐67 membranes.

As aforementioned, the BCP/ZIF‐67 membranes were soaked in the mixed solvent of toluene and methanol with a volume ratio of 35/65 for 3 s prior to ZIF‐67 growth. Under this condition, the pore size of the membranes was significantly smaller than that of the pure BCP membranes. When the volume ratio of toluene/methanol in the mixed solvents was decreased to 30/70, the surface of the resulting BCP/ZIF‐67 membranes was partially covered by ZIF‐67 (**Figure** **2**a), with narrowed pores averaging ≈50 nm in size (Figure 2e). When the volume ratio of toluene/methanol was increased to 40/60, the typical 2D structures were formed on the surfaces of the BCP/ZIF‐67 membranes (Figure 2b), with pore size determined to be ≈78 nm. Further increasing the volume ratios of toluene/methanol to 45/55 and 50/50 resulted in the BCP/ZIF‐67 membranes exhibiting numerous 2D lamellar crystals on the surfaces (Figure 2c,d), with the average pore sizes of ≈80 and 87 nm, respectively. We noted that the pore sizes of the membranes with in situ growth of 2D ZIF‐67 were smaller than those of the pure BCP membranes (Figures S7 and S8a, Supporting Information, and Figure 2e). This reduction in pore size can be attributed to two factors: first, the in situ growth of ZIF‐67, which constricts the pores of the BCP/ZIF‐67 membranes, and second, the stronger affinity of methanol for the P4VP of PS‐*b*‐P4VP BCP.^[^ ^22a^ ^]^ The quenching of the BCP membranes in methanol causes inevitable shrinkage when these membranes are later immersed in aqueous solutions of ZIF‐67 precursors, further tightening the pores. Consequently, the thicknesses of the BCP/ZIF‐67 membranes were measured to be 0.67, 1.06, 1.01, 0.98, and 0.96 µm for toluene‐to‐methanol volume ratios of 30/70, 35/65, 40/60, 45/55, and 50/50, respectively. These values are significantly lower than the corresponding values of pure BCP membranes (Figure S8b, Supporting Information and Figure 2f).

When the volume ratio of toluene/methanol was maintained at 35/65 in the mixed solvents, the BCP/ZIF‐67 membranes prepared by varying the soaking durations consistently exhibited porous structures (Figure S9a–d, Supporting Information). The average pore size of the hybrid membranes was determined to be 64 nm in the case where the soaking duration was 1 s (Figure S10a, Supporting Information). With extended soaking durations of 5, 7, and 9 s, the average pore sizes increased to 70, 74, and 77 nm, respectively. Both the porosity and pore size of the prepared BCP/ZIF‐67 hybrid membranes were significantly lower than those of the pure BCP membranes (Figure S9e–h, Supporting Information), consistent with the trends observed in the BCP/ZIF‐67 membranes fabricated using varying volume ratios of toluene and methanol in the mixed solvents. Furthermore, the thicknesses of the BCP/ZIF‐67 membranes were measured as 1.04, 1.06, 1.08, 1.09, and 1.07 µm for soaking durations of 1, 3, 5, 7, and 9 s, respectively (Figure S10b, Supporting Information). The lower thicknesses of the hybrid membranes compared to the pure BCP membranes can be attributed to the water‐induced shrinkage during the synthesis of 2D ZIF‐67. This observation aligns with the aforementioned analyses, further supporting the impact of the synthesis process on the structural characteristics of the hybrid membranes.

**Figure 3:**
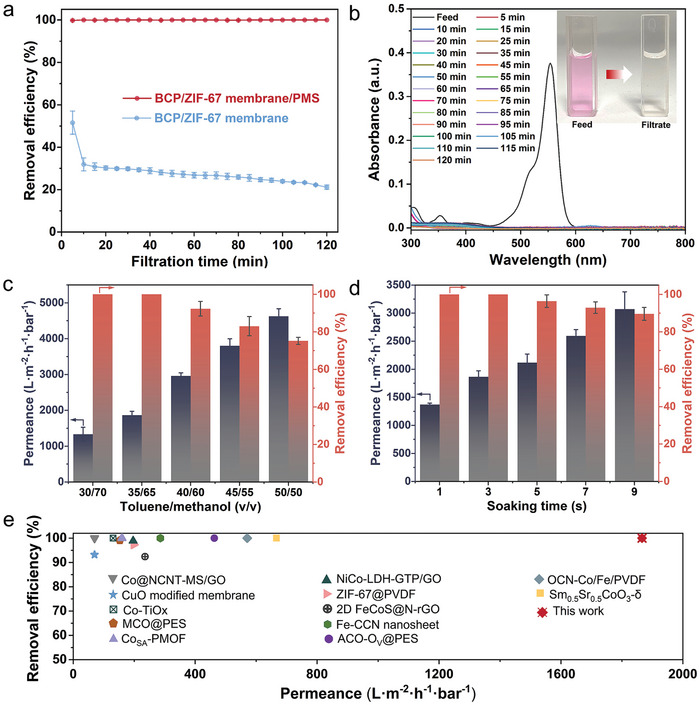
Catalytic performance of the BCP/ZIF‐67 membranes. a) Continuous dynamic catalytic processes of the BCP/ZIF‐67 membranes with and without PMS during 120 min of filtration. b) UV–vis spectra of the feed and filtrates of the aqueous solutions of RhB within 120 min of catalytic filtration. The catalytic membranes used for 120 min of filtration in (a) and (b) were prepared by soaking in mixed solvents of toluene and methanol with a volume ratio of 35/65. c) Histograms showing the permeance and removal efficiency of the BCP/ZIF‐67 membranes derived by soaking in mixed solvents containing toluene and methanol with different volume ratios for 3 s. d) Histograms showing the permeance and removal efficiency of the BCP/ZIF‐67 membranes derived by soaking in the mixed solvents of toluene and methanol with the volume ratio of 35/65 for various durations. e) Comparison of the catalytic performance of the BCP/ZIF‐67 membranes with other reported membranes.

The synthesized BCP/ZIF‐67 hybrid membranes, featuring the unique “2D MOFs within 3D membranes” structure, are highly promising for the efficient degradation of pollutants in water. Rhodamine B (RhB, 479.01 g mol^−1^), a typical electroneutral dye used in the textile industry, was selected as the model pollutant in this study. When aqueous solutions of RhB (pH = 7) were passed through the BCP/ZIF‐67 catalytic membranes, PMS activated by the 2D Co‐based ZIF‐67 anchored onto the nanopore walls of the hybrid membranes generated ROS for degrading RhB. The BCP/ZIF‐67 membranes demonstrated a stable degradation rate of 100% for RhB in the presence of PMS (**Figure** **3**a), highlighting their strong and durable catalytic degradation capability. Furthermore, the XPS wide‐scan spectra of the BCP/ZIF‐67 membranes after 120 min of filtration showed no noticeable differences compared to the pristine membranes (Figure S11, Supporting Information versus Figure 1g), confirming the robustness of the synthesized catalytic membranes for water treatment. In contrast, without PMS, the catalytic membranes exhibited an initial RhB elimination efficiency of only 52%, which sharply decreased to 32% within 10 min of filtration. Over the course of 120 min, the efficiency further declined gradually to 21%. This behavior indicates that, in the absence of PMS, adsorption‐not size sieving‐plays the dominant role in removing RhB, given that the average pore size of the BCP/ZIF‐67 catalytic membranes (≈70 nm) is much larger than the size of RhB molecules (≈1.8 nm).^[^ ^35^ ^]^ Therefore, in the absence of PMS, adsorption rather than size sieving predominantly governs the removal of RhB. This is attributed to the well‐known large surface areas of 2D MOFs,^[^ ^36^ ^]^ which enable high‐efficiency dye elimination through rapid saturation of adsorption sites over short periods. However, once these sites are saturated, the elimination efficiency declines sharply.

However, when RhB aqueous solutions containing PMS passed through the hybrid membranes, the confined nanopore space of the membranes restricted the free motion of RhB and PMS molecules within the solution. The 3D tortuous pore structures further promoted collisions between PMS and the catalytic sites of the 2D MOFs, enhancing PMS activation. Simultaneously, this confinement increased the interactions between the generated ROS and RhB molecules. As a result, RhB molecules, which were strongly adsorbed onto the membrane pore walls, were rapidly degraded by the activated PMS. This degradation released active sites for re‐adsorption, enabling a continuous cycle of adsorption and degradation. This mechanism facilitated ultrafast RhB degradation within the 3D nanoporous hybrid membranes, as illustrated in Figure 3b.

With increasing volume ratios of toluene/methanol in the mixed solvents, the produced BCP/ZIF‐67 membranes exhibited progressively higher permeance for the aqueous solutions of RhB, while the corresponding removal efficiencies decreased (Figure 3c). When the hybrid membranes fabricated using mixed solvents with a toluene‐to‐methanol volume ratio of 30/70, the permeance was 1335 L (m^2^·h·bar)^−1^ with a removal efficiency of 100%. Increasing the ratio to 35/65 improved the permeance to 1866 L (m^2^·h·bar)^−1^. This enhancement in permeance was primarily attributed to the widened pores (Figure 2e) and the enhanced hydrophilicity of the membranes (Figure S12a, Supporting Information). Despite the significant increase in membrane thickness from 0.67 to 1.06 µm (Figure 2f), which introduced longer catalytic pathways, the RhB removal efficiency remained at 100%. When the volume ratio of toluene/methanol in the mixed solvents was increased to 40/60, further enlargement of the membrane pores resulted in a permeance as high as 2958 L (m^2^·h·bar)^−1^. However, the removal efficiency of RhB slightly decreased to 92.3%. With further increases in the ratio to 45/55 and 50/50, the permeances of the catalytic membranes were significantly enhanced to 3800 and 4625 L (m^2^·h·bar)^−1^, respectively. However, the expanded catalytic spaces resulted in the reduced removal efficiencies, dropping to 83% and 75% for the respective membranes.

In addition to adjusting the volume ratios of toluene and methanol in the mixed solvents, the soaking time during BCP membrane fabrication can also be varied to finely tune the catalytic performance of the BCP/ZIF‐67 membranes (Figure 3d). When the volume ratio of toluene/methanol was fixed at 35/65, the resulting catalytic membranes displayed the permeances of 1371, 1866, 2117, 2593, and 3070 L (m^2^·h·bar)^−1^ for the soaking durations of 1, 3, 5, 7 and 9 s, respectively. Furthermore, the removal efficiencies of 100% for RhB were guaranteed when the soaking durations of 1 and 3 s were applied in fabricating the catalytic membranes. The removal efficiencies were capable of being retained as 96%, 92.9% and 90% even the soaking durations were further extended to 5, 7 and 9 s, respectively. The increased permeances were attributed to the widened pores and enhanced hydrophilicity induced by longer soaking durations (Figures S10a and S12b, Supporting Information), consistent with the trends observed in catalytic membranes prepared using varying toluene/methanol volume ratios in the mixed solvents. The slight reduction in RhB removal efficiency with increasing pore size was ascribed to the diminished effect of nano‐confined AOPs in larger membrane pores.

As the membrane pores offer the confined space for AOPs, it is well accepted that the narrowed pores smaller than 20 nm are in favor of high degradation efficiency.^[^ ^8^ ^]^ However, in this work, the membrane pores as large as ≈70 nm are still able to make 100% of RhB degradation available. The permeability‐degradation efficiency trade‐off was thus broken. Even the pores of the hybrid membranes were widened to ≈77 nm, 90% of RhB was degraded while a permeance of 3070 L (m^2^·h·bar)^−1^ was acquired. It is worth noting that the degradation efficiency of the hybrid membranes was still maintained as 75% accompanied with a high permeance of 4625 L (m^2^·h·bar)^−1^. The weakened pore confinement effect is ascribed to the combination the superior 2D ZIF‐67 catalysts with the 3D BCP membranes. 2D ZIF‐67 with abundant active sites can facilitate adsorption and further degradation of pollutants, while the BCP membranes offer the unique tortuous pore structures for extended catalytic channels. Nevertheless, the catalytic performance of the BCP/ZIF‐67 membranes, including both permeance and removal efficiency, significantly outperformed other reported catalytic membranes (Figure 3e, Table S1, Supporting Information). Compared to the state‐of‐the‐art catalytic membranes, the diminished pore confinement effect allows the widening of the pores of the BCP/ZIF hybrid membranes, thereby producing the higher permeances. This is the motivation of this work. However, the larger membrane pores may improve the dosage of PMS, further increasing the cost for treating wastewater.

**Figure 4:**
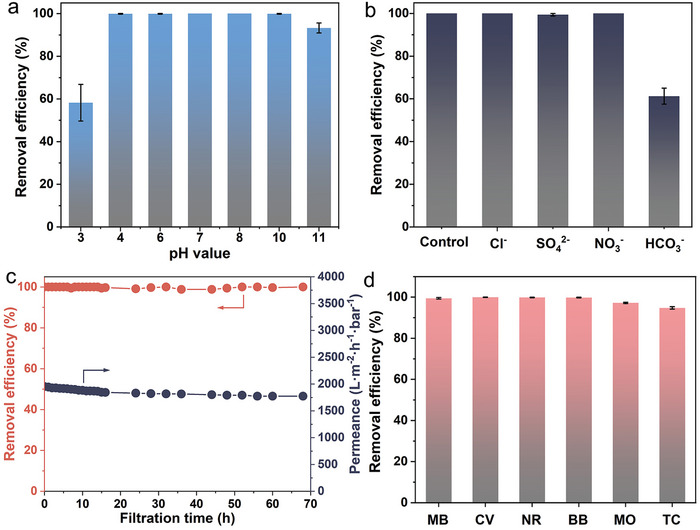
The stability of the BCP/ZIF‐67 membranes for catalytic degradation. Histogram showing the removal efficiency of the BCP/ZIF‐67 membranes for degrading RhB under different a) pH values or b) background anions. c) Dynamic removal efficiency of BCP/ZIF‐67 membranes during long‐term filtration. d) Histogram displaying the removal efficiency of the BCP/ZIF‐67 membranes for degrading a variety of pollutants.

In additional to the permeance and degradation efficiency, the stability of the hybrid membranes under varied and complex conditions is crucial for practical applications. Since actual wastewater often deviates from pH neutrality,^[^ ^37^ ^]^ aqueous solutions of RhB with pH values ranging from 3 to 11 were prepared to evaluate the degradation performance of the BCP/ZIF‐67 catalytic membranes. The hybrid membranes demonstrated exceptional stability, retaining nearly 100% removal efficiency for RhB over a broad pH range of 4 to 10 (**Figure** **4**a). This performance is markedly superior to other Co‐based catalytic membranes.^[^ ^10^, ^38^ ^]^ This excellent stability is primarily attributed to the durability of ZIF‐67, which is anchored onto the nanopore walls of the BCP membranes. ZIF‐67 is a well‐known MOF with outstanding resistance to acidic and alkaline environments.^[^ ^39^ ^]^ At pH 11, the degradation efficiency of the hybrid membranes remained high at 94%, while it dropped to 58% at pH 3. It has been reported that the crystal structure of ZIF‐67 can be partially degraded under strongly acidic (pH 3) and strongly alkaline (pH 11) conditions.^[^ ^40^ ^]^ The diminished degradation efficiency at pH 3 can also be attributed to the protonation of the BCP, which stretches the hydrophilic P4VP chains coordinated with ZIF‐67, disrupting the grown ZIF‐67 structure. Hence, the degradation ability of the catalytic membranes in strongly acidic solutions (pH 3) was significantly weakened. This can be attributed to the protonation of the BCP, which caused the hydrophilic P4VP chains coordinated with ZIF‐67 to stretch under acidic conditions, ultimately disrupting the structure of the grown ZIF‐67. However, in strongly alkaline solutions of RhB (pH 11), the structural stability of ZIF‐67 was partially preserved due to its strong coordination with the P4VP chains. This coordination likely contributed to maintaining relatively high degradation efficiency despite the partial degradation of ZIF‐67 under such conditions.

To address the complex components of actual wastewater, various anions, including Cl^−^, SO_4_^2−^, NO_3_^−^ and HCO_3_^−^, were introduced into RhB aqueous solutions to evaluate their impact on the degradation performance of the BCP/ZIF‐67 catalytic membranes. These anions are known to inhibit PMS‐induced degradation processes.^[^ ^10b^ ^]^ The catalytic membranes maintained nearly 100% removal efficiency for RhB, even in the presence of Cl^−^, SO_4_^2−^ or NO_3_^−^ (Figure 4b), demonstrating their strong resistance to the inhibitory effects of these anions. However, HCO_3_^−^, which raises the solution's pH and simultaneously consumes ROS during PMS‐induced degradation,^[^ ^10b^ ^]^ reduced the degradation efficiency to 61%. This performance remains comparable to other reported works.^[^ ^12^ ^]^

The stability of the BCP/ZIF‐67 catalytic membranes was further evaluated by the long‐term degradation of RhB (Figure 4c). The membranes consistently maintained a near 100% degradation efficiency for RhB, with a highly stable permeance of ≈1800 L (m^2^·h·bar)^−1^ during 68 h‐continuous operation. This performance underscores the robust nature of the synthesized catalytic membranes, which is superior in comparison with other findings (Table S2, Supporting Information). Moreover, the concentration of cobalt ions in the filtrate was determined as low as 2.35 µg L^−1^, significantly below the threshold concentration suggested by the U.S. Environmental Protection Agency for reclaimed water (≤50 µg·L^−1^). To demonstrate the versatility of the BCP/ZIF‐67 catalytic membranes, their performance was evaluated against a range of simulated contaminants, including positively charged dyes (methylene blue (MB), crystal violet (CV), and neutral red (NR)) and negatively charged dyes (brilliant blue (BB) and methyl orange (MO)) (Figure 4d). The BCP/ZIF‐67 membranes effectively degraded 100% of MB, CV, NR, and BB, and 97% of MO within 120 min of continuous filtration, suggesting the excellent catalytic degradation stability of the synthesized catalytic membranes for various organic pollutants. Additionally, the catalytic membranes can remove 95% of tetracycline (TC), which is much higher than the conventional PMS‐based degradation by ZIF‐67.^[^ ^16b^ ^]^

To demonstrate the importance of 2D ZIF‐67 of the catalytic membranes in degrading pollutants, the hybrid membrane composed of 3D ZIF‐67 and a BCP membrane was further synthesized. The cubic crystals with varied sizes dwelled on the membrane surface unambiguously (Figure S13a, Supporting Information), implying the success synthesis of 3D ZIF‐67 in the BCP membrane. The average pore size of this hybrid membrane was assigned as ≈25.3 nm, which is much smaller than that of the aforementioned BCP/ZIF‐67 membrane (≈70 nm). The narrowed pores were ascribed to the fast growth of 3D ZIF‐67 in the BCP matrix membrane, which was revealed by the cross section of the membrane with sparse pores (Figure S13b, Supporting Information). The aggressive growth of 3D ZIF‐67 made the thickness of the produced membrane slightly increase to ≈987 nm as compared to the BCP/ZIF‐67 membrane (≈828 nm). Although the membrane constructed from 3D ZIF‐67 degraded RhB with an efficiency of almost 100% (Figure S14, Supporting Information), both the permeance and its stability were much lower than that of the BCP/ZIF‐67 membrane. Therefore, 2D ZIF‐67 rather than 3D ZIF‐67 can help prepare high‐performance catalytic membranes for AOPs.

**Figure 5:**
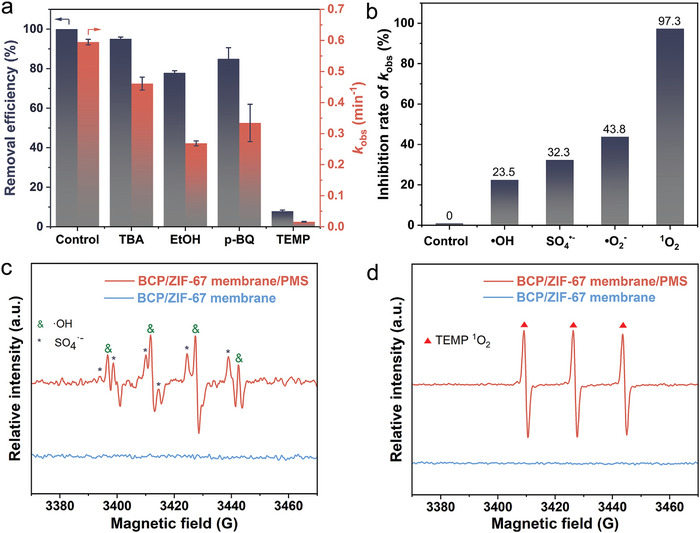
Identification and contribution of ROS generated in the BCP/ZIF‐67 membrane/PMS system. a) Histogram of removal efficiency and *k*_obs_ of the BCP/ZIF‐67 membrane/PMS system for degrading RhB in the presence of TBA, EtOH, p‐BQ or TEMP. b) Inhibition rate constant *k*_obs_ of various scavengers. c) Identification of the radicals of SO_4_^·−^ and ·OH using 5,5‐dimethyl‐pyrroline‐N‐oxide (DMPO, 100 mM) as a spin‐trapping agent. d) Detection of ^1^O_2_ by employing TEMP (50 mM). [Correction added on 20 February 2025, after first online publication: figure 5 image is replaced.]

To elucidate the mechanism of catalytic degradation confined within the nanopores of the BCP/ZIF‐67 membranes, the ROS generated during the PMS‐based membrane process were analyzed using specific quenchers: *tert*‐butyl alcohol (TBA), ethanol (EtOH), p‐benzoquinone (p‐BQ), and 2,2,6,6‐tetramethyl‐4‐piperidinol (TEMP). These quenchers were employed to evaluate the contributions of ·OH, ·OH/SO_4_^·−^, O_2_^·−^, and ^1^O_2_ respectively (**Figure** **5**a). When RhB aqueous solution mixed with TBA passed through the BCP/ZIF‐67 membranes, the removal efficiency of RhB slightly decreased from 100% to 95%, indicating the presence of small amounts of ·OH in the BCP/ZIF‐67 membrane/PMS system. Similarly, the use of p‐BQ, which targets O_2_^·−^ resulted in only a minor reduction in RhB removal efficiency to 85%, suggesting an insignificant role for O_2_^·−^ in the catalytic degradation process. When EtOH, a quencher for both ·OH and SO_4_^·‐^ was employed, the removal efficiency of RhB decreased to 78%. By comparing the quenching effects of TBA and EtOH, the noticeable contribution of SO_4_^·‐^ to the catalytic degradation was identified. Notably, when TEMP, a scavenger for ^1^O_2_, was introduced, the removal efficiency of RhB dramatically declined to just 8%. This observation highlights the critical role of ^1^O_2_ in the catalytic degradation process within the BCP/ZIF‐67 membrane/PMS system.

The quencher‐induced reduction in the degradation capability of the BCP/ZIF‐67 membrane/PMS system was further confirmed by the first‐order kinetic rate constant (*k*_obs_). The presence of TEMP caused a substantial decrease in the *k*_obs_ from 0.595 min^−1^ to 0.016 min^−1^, indicating that TEMP had the strongest quenching effect among the tested quenchers. This confirms that the non‐radical ^1^O_2_ is the predominant ROS responsible for the catalytic degradation of RhB in the BCP/ZIF‐67 membrane/PMS system. To better understand the roles of radical ROS (·OH, SO_4_^·−^, O_2_^·−^) and non‐radical ROS (^1^O_2_) in the catalytic degradation process, the inhibition rates of *k*_obs_ were calculated (Figure 5b). The contribution of radical and non‐radical ROS to the catalytic degradation in the developed BCP/ZIF‐67 membrane/PMS system was compared as follows: ^1^O_2_ (97.3%) > ·O_2_^·−^ (43.8%) > SO_4_^·−^ (32.3%) >·OH (22.5%). These findings emphasize the critical role of ^1^O_2_ in ensuring the durability and high catalytic efficiency of the BCP/ZIF‐67 membrane/PMS system. However, it is worth noting that dynamic interactions and conversions between different reactive species may occur during the catalytic degradation process, further influencing the overall performance.^[^ ^41^ ^]^

Electron paramagnetic resonance (EPR) spectroscopy was subsequently performed to clarify the ROS species during catalytic degradation. The spin‐trapping agent 5,5‐dimethyl‐pyrroline‐*N*‐oxide (DMPO) was used to capture SO_4_^·−^ and ·OH radicals (Figure 5c), while TEMP was used to trap ^1^O_2_ (Figure 5d). In the absence of PMS, no peaks were detected in the EPR spectrum of the BCP/ZIF‐67 membranes, confirming the lack of ROS generation under these conditions. In contrast, when PMS was introduced, the EPR spectrum of the BCP/ZIF‐67 membrane/PMS system revealed characteristic signals corresponding to both SO_4_^·−^ and ·OH radicals in the presence of DMPO. The intensities of the peaks derived from DMPO‐SO_4_^·−^ were weaker than those of the DMPO‐·OH adducts, likely due to the rapid nucleophilic substitution reaction that converts DMPO‐ SO_4_^·−^ into DMPO‐·OH. Furthermore, the characteristic triplet peaks (1:1:1) of ^1^O_2_ were also detected in the BCP/ZIF‐67 membrane/PMS system. These findings from EPR spectroscopy align closely with the results of the quenching experiments, confirming the coexistence of multiple ROS species and further highlighting the dominant role of ^1^O_2_ in catalytic degradation.

**Figure 6:**
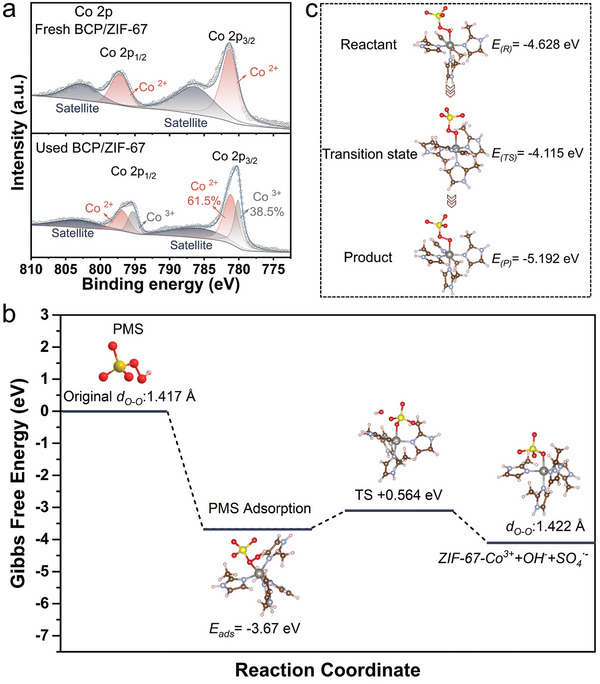
a) Co 2p XPS spectra of the BCP/ZIF‐67 membranes before (fresh) and after (used) 2 h of continuous degradation. b) The bond lengths of the original and activated O‐O bond length (*d*_O‐O_) in the PMS molecule, along with the electron density difference. c) The electron density difference during the redox transformation from Co^3+^ to Co^2+^ when HSO_5_^−^ is oxidized to SO_5_^·−^ by Co^3+^.

To gain deeper insights into the degradation mechanism of the BCP/ZIF‐67 membrane/PMS system, the surface compositions of the BCP/ZIF‐67 membrane were analyzed using XPS before (fresh) and after (used) dynamic filtration (**Figure** **6**a). Two new peaks at 780.1 eV and 795.3 eV were observed in the spectrum of the used membrane, corresponding to Co^3+^, indicating that 38.5% of the original Co^2+^ has been converted to Co^3+^ during the catalytic degradation process. This transformation can be attributed to the reaction between Co^2+^ and HSO_5_^−^, which oxidizes Co^2+^ to Co^3+^ while producing SO_4_^−·^ through electron transfer (Equation (1)).^[^ ^42^ ^]^ Simultaneously, Co^3+^ can be reduced back to Co^2+^ by HSO_5_^−^ (Equation (3)).^[^ ^10c^ ^]^ This dynamic redox cycling of Co ions underscores the ability of the BCP/ZIF‐67 membranes to facilitate efficient electron transfer and rapid regeneration of active catalytic sites. These features are critical for the high‐efficiency elimination of pollutants from aqueous solutions, enabling sustained catalytic performance over extended filtration periods.

Density functional theory (DFT) calculations were also performed to reveal the catalytic mechanism of the BCP/ZIF‐67 membrane/PMS system (Figure 6b). The adsorption energy (E_ads_) of the PMS molecule on the surface of the BCP/ZIF‐67 membrane was computed to be ‐3.67 eV, which is higher than the E_ads_ of PMS adsorbed on the 3D ZIF‐67 surface.^[^ ^43^ ^]^ This indicates that the spontaneous dissociation of PMS during the adsorption of PMS molecules in the BCP/ZIF‐67 membrane would be accelerated.^[^ ^44^ ^]^ Moreover, the O‐O bond length (*d*_O‐O_) within the PMS molecule was expanded from 1.417 to 1.422 Å after adsorption onto the surface of the BCP/ZIF‐67 membranes. The cleavage of the O‐O bond was thus facilitated, thereby activating the HSO_5_^−^ group to produce ·OH and SO_4_^·−^ (Equation (1) and (2)).^[^ ^41^, ^45^ ^]^ Subsequently, the system produced O_2_^·−^ as a result of the reaction between ·OH and H_2_O_2_ (Equation ((4), (5), (6))).^[^ ^46^ ^]^ These persistently generated free radicals can continuously attack the organic pollutant molecules, leading to their ongoing degradation.

(1) $$Co^{2+}+HS{O_{5}}^{-}\to Co^{3+}+OH^{-}+S{O_{4}}^{\cdot -}$$

(2) $$S{O_{4}}^{\cdot -}+H_{2}O\to S{O_{4}}^{2-}+\cdot OH+H^{+}$$

(3) $$Co^{3+}+HS{O_{5}}^{-}\to Co^{2+}+H^{+}+S{O_{5}}^{\cdot -}$$

(4) $$HS{O_{5}}^{-}+H_{2}O\to H_{2}O_{2}+HS{O_{4}}^{-}$$

(5) $$\cdot OH+H_{2}O_{2}\to H{O_{2}}^{\cdot -}+H_{2}O$$

(6) $$H{O_{2}}^{\cdot -}\to H^{+}+{O_{2}}^{\cdot -}$$

(7) $$HS{O_{5}}^{-}\to S{O_{5}}^{\cdot -}+H^{+}+e^{-}$$

(8)

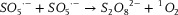



(9)

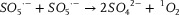




The electron density difference of the transformation from Co^3+^ to Co^2+^ further revealed the free energy of −4.628, −4.115 and −5.192 eV for the reactant, transition state and product, respectively (Figure 6c). The oxidation of HSO_5_^−^ to SO_5_^·−^ by Co^3+^ was accompanied by the reduction of Co^3+^ to Co^2+^ (Equation (3)). The resultant reaction energy (Δ*E*) was calculated to be ‐0.564 eV (Equation (6), Supporting Information). The transformation of Co^3+^ to Co^2+^ was thus thermodynamically favorable, and the conversion and regeneration within the Co^2+^/Co^3+^ cycle are competent in furnishing the continuous generation of ROS. Additionally, the adsorption of PMS onto the 2D ZIF‐67 structures grown on the membrane pore walls caused a significant increase in the O‐H bond length of PMS, from 0.974 Å to 0.986 Å. This elongation suggests bond disruption and the detachment of the hydrogen atom from PMS. During this reaction, PMS acted as an electron donor, emitting electrons and forming the PMS anion radical (SO_5_^·−^) (Equation (7)). Due to the high reaction rate and low activation energy, the self‐reaction of SO_5_^·‐^ progressed rapidly (Equations (8) and (9)). This reaction led to the formation of S_2_O_8_^2−^, SO_4_^2−^ ions, accompanied by the release of ^1^O_2_.^[^ ^47^ ^]^

Therefore, the PMS‐activated catalytic degradation of organic pollutants confined in the BCP/ZIF‐67 membranes can be proposed as follows: When PMS is adsorbed on the Co‐N sites of 2D ZIF‐67, electrons are quickly transferred to PMS, promoting the generation of ·OH and SO_4_^·−^.^[^ ^48^ ^]^ Meanwhile, PMS adsorbed on the 2D ZIF‐67 surface donates electrons via O‐H bond cleavage, resulting in the formation of SO_5_^·−^. The generated SO_5_^·‐^ interact with each other, leading to the production of ^1^O_2_ on the surface and within the nanopore walls of the BCP/ZIF‐67 membranes. Consequently, the produced radicals (SO_4_^·−^, ·OH, ·O_2_^−^) and non‐radicals (^1^O_2_) attacked and decomposed organic pollutants in the nanopores of the BCP/ZIF‐67 membranes. The unique architecture of the BCP membranes, combined with the high surface area of the 2D ZIF‐67, enhances the efficiency of pollutant degradation. This is achieved through strong adsorption capability, abundant catalytic active sites, and extended catalytic channels that optimize the PMS‐induced ^1^O_2_ generation and pollutant degradation.

## 3 Conclusion

The robust BCP/ZIF‐67 hybrid membranes were successfully fabricated by the in situ growth of 2D ZIF‐67 onto the nanopore walls of 3D BCP membranes, enabling ultrafast molecular degradation. These membranes demonstrated outstanding performance, continuously degrading 100% of RhB from aqueous solutions while maintaining a permeance greater than 1800 L (m^2^ **·**bar**·**h)^−1^, surpassing previously developed membranes. The hybrid membranes' performance stems from their precisely tuned pore size, thickness, and hydrophilicity, which allowed for adjustable permeance and degradation efficiency. Even at a permeance as high as 3070 L (m^2^ **·**bar**·**h)^−1^, the degradation efficiency remained at 90%. Additionally, the membranes exhibited excellent resistance to both acidic and alkaline conditions and maintained superior catalytic performance in the presence of various background anions. Long‐term catalytic filtration was ensured by the stability of the hybrid membranes and the generation of the non‐radical species ^1^O_2_. The degradation mechanism was further elucidated through DFT simulations, providing a deep understanding of the catalytic process. This study introduces an innovative approach for designing advanced catalytic membranes, enabling the rapid and continuous high‐efficiency degradation of small molecules, paving a way for practical applications in water treatment.

## Conflict of Interest

The authors declare no conflict of interest.

## Supporting information



Supporting Information

## Data Availability

The data that support the findings of this study are available on request from the corresponding author. The data are not publicly available due to privacy or ethical restrictions.
