# Supplementary material for: In Situ Growth of Robust 2D ZIF‐67 MOF in Block Copolymer Membranes for Ultrafast Molecular Degradation
Source: Adv Sci (Weinh). 2025 Feb 18;12(14):2416169. doi: 10.1002/advs.202416169 (PMC11984913; doi:10.1002/advs.202416169)
Supplement: Supplementary file 1 — Supporting Information [file ADVS-12-2416169-s001.docx]

*Supporting Information for*

**In-situ growth of robust 2D ZIF-67 in block copolymer membranes for ultrafast molecular degradation**

Jingjing Xu,^a^ Jianyong Yu,^b^ Leiming Guo,^a,*^ Faxue Li,^a,*^ and Nikos Hadjichristidis^c^

^a^ Key Laboratory of Textile Science & Technology, Ministry of Education, College of Textiles, Donghua University, Shanghai 201620, China

^b^ Innovation Center for Textile Science and Technology, Donghua University, Shanghai 201620, China

^c^ Polymer Synthesis Laboratory, KAUST Catalysis Center, Physical Science and Engineering Division, King Abdullah University of Science and Technology (KAUST), Thuwal 23955, Saudi Arabia

^*^ Email: leiming.guo@dhu.edu.cn; fxlee@dhu.edu.cn

**Experimental Section**

**Materials.** Polystyrene-*block*-poly (4-vinylpyridine) (PS-*b*-P4VP, *M*_n_^PS^=120 kg/mol, *M*_n_^P4VP^=20 kg/mol, *Ð*=1.18) was purchased from Polymer Source (Canada). Chloroform (>99.8%) was supplied by Shanghai Titan Technology Co. Ltd. (Shanghai, China). Methanol (>99.8%), toluene (>99.8%), Sodium hydroxide (NaOH, >96.0%), sodium chloride (NaCl, >99.5%), sodium nitrate (NaNO_3_, >99.0%), sodium sulfate (Na_2_SO_4_, >99.0%), sodium bicarbonate (NaHCO_3_, >99.5%), hydrochloric acid (HCl, 36.0%~38.0%) were obtained from Sinopharm Group (Shanghai, China). 30 wt% aqueous solution of poly (methacrylic acid, sodium salt) (NaPMA, *M*n=5400 g/mol, *M*_w_=9500 g/mol) was received from Sigma Aldrich (USA). Cobalt nitrate hexahydrate (Co(NO_3_)_2_·6H_2_O, >99.0%), tert-butyl alcohol (TBA, >99.0%), p-benzoquinone (p-BQ, >99.0%), 5,5-Dimethyl-pyrroline-N-oxide (DMPO, >97%), Acid Red 73 (AR, >100%) were obtained from Macklin (Shanghai, China). Potassium monopersulfate triple salt (PMS, KHSO_5_) and 2,2,6,6-tetramethyl-4-piperidinol (TEMP, >98.0%) were supplied by Shanghai Aladdin Biochemical Technology Co., Ltd. (Shanghai, China). 2-methylimidazole (2-MI) was bought from Shanghai Haohong Scientific Co., Ltd. (Shanghai, China). Rhodamine B (RhB, >95%) were bought from Meryer Biochemical Technology Co., Ltd. (Shanghai, China). Methylene blue (MB, >98%) was purchased from Yinokai Technology Co., Ltd (Beijing, China). Crystal violet (CV, >90%), Methyl orange (MO, >98%), Neutral red (NR, >98%), which were all obtained from Shanghai Bide Pharmaceutical Technology Co., Ltd (Shanghai, China). All chemicals were used as received without further purification after receiving. Purified water used throughout the experiments having a resistance > 18.2 MΩ cm^2^ in this work was produced by the purification system of Merck.

The microscopy slides (size: 25.4 × 76.2 mm; thickness: 1.1 ± 0.1 mm) and round-shaped silicon wafers (diameter: 76.2 ± 0.33 mm; thickness: 380 ± 20 µm) used for substrates were purchased from Yancheng Feizhou Glass Co., Ltd. and Luva System Inc., respectively. The silicon wafers were cut into small pieces of 2 cm × 2 cm, and then ultrasonically cleaned twice in acetone and ethanol sequentially for about 15 min each time before use. Microscope slides were cut to 3 cm × 3 cm and cleaned in the same way as silicon wafers were handled to serve as substrates for the preparation of composite membranes. Hydrophilic polyvinylidene fluoride (PVDF) with a diameter of 25 mm and an average diameter of 0.22 µm was obtained from Millipore as a matrix membrane for the preparation of composite membranes.

**Generation of porous structures of PS-*b*-P4VP membranes on silicon wafers.** PS-*b*-P4VP copolymers were dissolved in chloroform at room temperature (rt, 25℃) to yield a 2 wt% polymer solution, which was then passed through PTFE filters with a pore size of 0.45 μm to remove large aggregates that may not be dissolved in the solution. Subsequently, a 150 µL solution was dropped onto a silicon wafer and spun onto the silicon wafers by a spin coater (KW-41/5, IMECAS) at a rotation speed of 3000 rpm and a rotation time of 30 s. The as-prepared membranes were immersed into the mixed solvents of methanol and toluene with different volume ratios for different durations, subsequently these membranes were taken out from the mixed solvents and immediately transferred into methanol for arbitrary durations. As a quenching solvent, methanol terminated the highly swollen state during the membranes sucked up the mixed solvents and avoided the happening of dissolution. Whereafter, the quenched membranes were air-dried naturally for further characterization, the membrane porosity (*P*) could be determined by equation (1):

$$\begin{aligned} P=\frac{T-T_{0}}{T}\times100\%\#\left( 1 \right) \end{aligned}$$

Where *T* and $\text{T}_{\text{0}}$ is the thickness of the nanoporous film and pristine film, respectively.

**Preparation of solid PS-*b*-P4VP membranes.** The process for preparation of the PS-*b*-P4VP membrane was based on our previous study. Briefly, 75 μL diluted aqueous solution of NaPMA with a concentration of 15wt% was dropped onto the glass slides, the PVDF membrane was then carefully placed on the surface of the NaPMA solution, and the solution rapidly diffused from the bottom of the PVDF membrane into the macropores due to the capillary force. After waiting for about 5 min at rt, the PVDF adhered tightly to the glass slides due to the high viscosity of NaPMA. Afterwards, 450 μL PS-*b*-P4VP solution was then dropped onto the PVDF membrane, followed spun by a spin coater at a spin velocity of 3000 rpm and a spin time of 30 s. The as-prepared composite membranes with a nonporous BCP layer and an underlying macroporous PVDF support were further immersed in water to remove the filled NaPMA, thus separating the composite membrane from the glass slide.

The composite membranes were then heat-treated in a vacuum oven at 110℃ under a surrounding pressure of 33.6 kPa for 20 min to enhance the affinity between the PS-*b*-P4VP layers and the PVDF supports. The oven temperature was then gradually decreased to rt.

**Preparation of BCP/ZIF-67 catalytic membrane.** The BCP/ZIF-67 membranes were synthesized using the in-situ synthesis method.^[1]^ First, the solid PS-*b*-P4VP films were immersed in the mixed solvent of toluene and methanol (V_toluene_/V_methanol_=30/60, 35/65, 40/60, 45/55, and 50/50) for 3 s, followed which the films were instantly taken out from the mixed solvent and further soaking in methanol. After ca. 2 min of soaking in methanol, the produced PS-*b*-P4VP membranes were transferred into 50 mL of aqueous solution consisted of Co(NO_3_)_2_·6H_2_O (0.03 M) and 2-MeIM (0.2 M) for 1 h at 25 ℃. The resulting membranes were thoroughly washed with deionized water and further dried at 25℃ for ca. 12 h, which were assigned as the BCP/ZIF-67 membranes derived by soaking in mixed solvents containing toluene and methanol with different volume ratios.

Once the solid PS-*b*-P4VP films were immersed in the mixed solvent of toluene and methanol (V_toluene_/V_methanol_=35/65) for varied durations (1, 3, 5, 7, and 9 s) followed by the same procedures as aforementioned, the BCP/ZIF-67 membranes derived by soaking in the mixed solvents of toluene and methanol with the volume ratio of 35/65 for various durations were then prepared.

To reveal the advantages of 2D ZIF-67 of the BCP/ZIF-67 catalytic membranes in degradation, the catalytic membrane composed of a BCP matrix membrane and 3D ZIF-67 was further synthesized. The synthesis of such catalytic membrane followed the same procedures as those employed for the preparation of the BCP/ZIF-67 membrane, with the exception that methanol was used for dissolving Co(NO_3_)_2_·6H_2_O (0.03 M) and 2-MeIM (0.2 M). Notably, the BCP membrane was produced by soaking the solid PS-*b*-P4VP film into toluene/methanol (V:V= 35/65) for 3 s followed by quenching in methanol.

**Characterizations.** The morphologies of PS-*b*-P4VP and BCP/ZIF-67 membrane were observed by field emission scanning electron microscope (FESEM, Hitachi SU8010, Japan). The cross-sectional element distribution of the BCP/ZIF-67 membrane was detected by energy dispersive spectrometer (EDS, Bruker QUANTAX, Germany). The X-ray diffraction patterns of both PS-*b*-P4VP and BCP/ZIF-67 membranes were characterized using a D8 advance X-ray diffractometer (XRD, Bruker, Germany) equipped with Cu Kα radiation. The measurements were conducted at a scanning rate of 5°/min over a 2θ range of 5° to 80°. Attenuated total reflection Fourier transform infrared spectroscopy (ATR-FTIR, PerkinElmer Spectrum two, UK) was performed to investigate the chemical structures of the PS-*b*-P4VP and BCP/ZIF-67 membranes. X-ray Photoelectron spectra (XPS, Thermo ESCALAB250, USA) were used to characterize the surface element chemical states of the PS-*b*-P4VP and BCP/ZIF-67 membranes. The water contact angle (WCA) was employed to evaluate the hydrophilicity of the prepared PS-*b*-P4VP and BCP/ZIF-67 membrane by sessile drop method (Biolin/Attension, KINO SL200KS, USA), in which the volume of water drop was set as 5 μL. The electronic universal tensile tester (MTS Exceed E42, China) was adopted to test mechanical strength of the PVDF support membrane, the BCP membrane, and the BCP/ZIF-67 membrane. The BCP membrane was produced by soaking the solid BCP film into toluene/methanol (V:V= 35/65) for 3 s followed by quenching in methanol, which was further used for preparing the BCP/ZIF-67 membrane. A sample with a size of 2.5 cm (L) × 0.5 cm (W) was stretched at a speed of 5 mm/min. The identification of active species generated during the catalytic processes was conducted using an electron paramagnetic resonance (EPR) spectrometer (Bruker EMXnano, German). To manifest the catalytic properties BCP/ZIF-67 membrane, the concentration of dye before and after the BCP/ZIF-67 membrane was monitored using UV-Vis spectrometer (Thermo EVOLUTION ONE, USA).

**Membrane performance testing**. Performance for the BCP/ZIF-67 membrane was evaluated using a home-made device with an effective membrane area of 1.77 cm^2^ (Figure S15). Prior to testing, the membranes were pre-pressurized with deionized water for 0.5 h to obtain a steady water flux. All the experiments were performed at 0.1 bar at rt. During the catalytic oxidation experiment, the feed solution was composed of 10 mg/L dye and 0.1 g/L PMS. Samples were collected at given time intervals and assessed the time course RhB degradation. A UV-vis spectrophotometer was utilized to measure the dye concentrations. The permeance (*J*) and dye removal efficiency (*R*) was calculated based on the following equations (2) (3):

$$\begin{aligned} J=\frac{V}{A\times t\times p}\#\left( 2 \right) \end{aligned}$$

$$\begin{aligned} R=\frac{{C_{0}-C}_{t}}{C_{0}}\times100\%\#\left( 3 \right) \end{aligned}$$

Where *V* is the volume of permeated dye filtrate (L), *t* is the permeation time (s), *A* is the membrane effective aera (m^2^), *p* is the transmembrane pressure. $\text{C}_{\text{0}}$ represents the initial dye concentration in the feed solution, while $\text{C}_{\text{t}}$ denotes the concentration of the sample solutions collected at specific time intervals.

In addition, the effects of solution pH (3 to 11) and background anions (SO_4_^2-^, Cl^-^, NO_3_^-^ and HCO_3_^-^) on dye degradation by the BCP/ZIF-67 membrane/PMS system were elucidated. HCl and NaOH were used to adjust the pH values of these solution. Hence, the general applicability of the BCP/ZIF-67 membrane/PMS system was evaluated by estimating the degradation efficiency of several dyes including methyl blue (MB), crystal violet (CV), methyl orange (MO), brilliant Blue (BB) and neutral red (NR). All experiments were repeated 3 times at least, and the average was presented.

**Quenching experiment.** To determine the role of hydroxyl radicals (·OH), sulfate radicals (SO_4_^.-^), singlet oxygen (^1^O_2_) and superoxide ion radicals (·O_2_^-^) in the dye degradation process, quenching experiments were performed using TBA (500 mM), ethanol (500 mM), TEMP (5 mM) and p-BQ (5 mM), separately. Normally, radical scavengers were added into the mixed solution of Rh B (10 mg/L) and PMS (0.1g/L) respectively, and then the solution was filtrated through the BCP/ZIF-67 membrane. The permeate was detected using UV-Vis spectrometer to quantify the contribution of multifarious radicals. Additionally, the intensity of these reactive species was measured using EPR spectroscopy (Bruker EMXnano, Germany). The first-order kinetic rate constant *k*_obs_ was calculated based on the equations (4):

$$\begin{aligned} \ln\left( \frac{C_{t}}{C_{0}} \right)=-k_{obs}\times t\#\left( 4 \right) \end{aligned}$$

Where $\text{C}_{\text{0}}$ and $\text{C}_{\text{t}}$ represent the initial RhB concentration and the concentration of the sample solutions collected at specific time intervals, respectively, and $\text{k}_{\text{obs}}$ denotes the fitted rate constant of pseudo first-order kinetic.

**Computational Methods.** All calculations were conducted employing the projector augmented wave (PAW) method within the framework of density functional theory (DFT), as implemented in the Vienna Ab initio Simulation Package (VASP).^[2]^ The electron exchange-correlation interactions were described using the generalized gradient approximation (GGA) with the Perdew-Burke-Ernzerhof (PBE) functional. PBE functional with D3(BJ) (DFT-D3 method with Becke-Jonson damping)^[3]^ dispersion correction was used to make sure that both the coulombic and Van der Waals interaction were decently interpreted during the whole process. The PAW method was utilized to model interactions between ion cores and valence electrons.^[4]^ Spin polarization effects were explicitly incorporated in the calculations. For geometry optimization, a plane-wave energy cutoff of 450 eV along with a Γ-centered k-mesh with a resolution of 2π × 0.04 Å⁻¹ was used to ensure the accurate calculation of energy.^[5]^ -0.02 eV/Å of geometry convergence condition was employed to ensure that the structures were correctly optimized to their corresponding state (ground state or transition state).^[6]^ A convergence of 10⁻⁵ eV during SCF was utilized to ensure a stable convergence of wavefunction and accurate gradient during optimization.^[7]^ 15 Å vacuum layer was built along Z axis to ensure the periodicity of X and Y direction, while the symmetry of the 2D ZIF-67 structure along Z axis was broken.^[8]^ Optimized structures were visualized by the VESTA software,^[9]^ while the VASPKIT code facilitated the pre- and post-processing of computational results.^[10]^ The Climbing Image Nudged Elastic Band (CI-NEB) method was employed to compute the corresponding transition states TS1 and TS2. The adsorption energies (*E_ads_*) of PMS and 2D ZIF-67 molecules were calculated by the following equation^[11]^:

$$\begin{aligned} E_{ads} =E_{\left( PMS+ZIF-67 \right)}-E_{PMS}-E_{ZIF-67}\#\left( 5 \right) \end{aligned}$$

Where, $\text{E}_{\text{(PMS+ZIF-67)}}$, $\text{E}_{\text{PMS}}$, and $\text{E}_{\text{ZIF-}\text{67}}$ represent the total energies of adsorption system, PMS molecule and 2D ZIF-67 surface, respectively.

The reaction energy ($\text{∆E}$) is defined as follows:

$$\begin{aligned} \Delta E=E_{\left( P \right)}-E_{\left( R \right)}\#\left( 6 \right) \end{aligned}$$

Where $\text{E}_{\text{(P)}}$ is the energy of the product in each reaction, $\text{E}_{\text{(R)}}$ is the energy of the reactant in reaction.

**
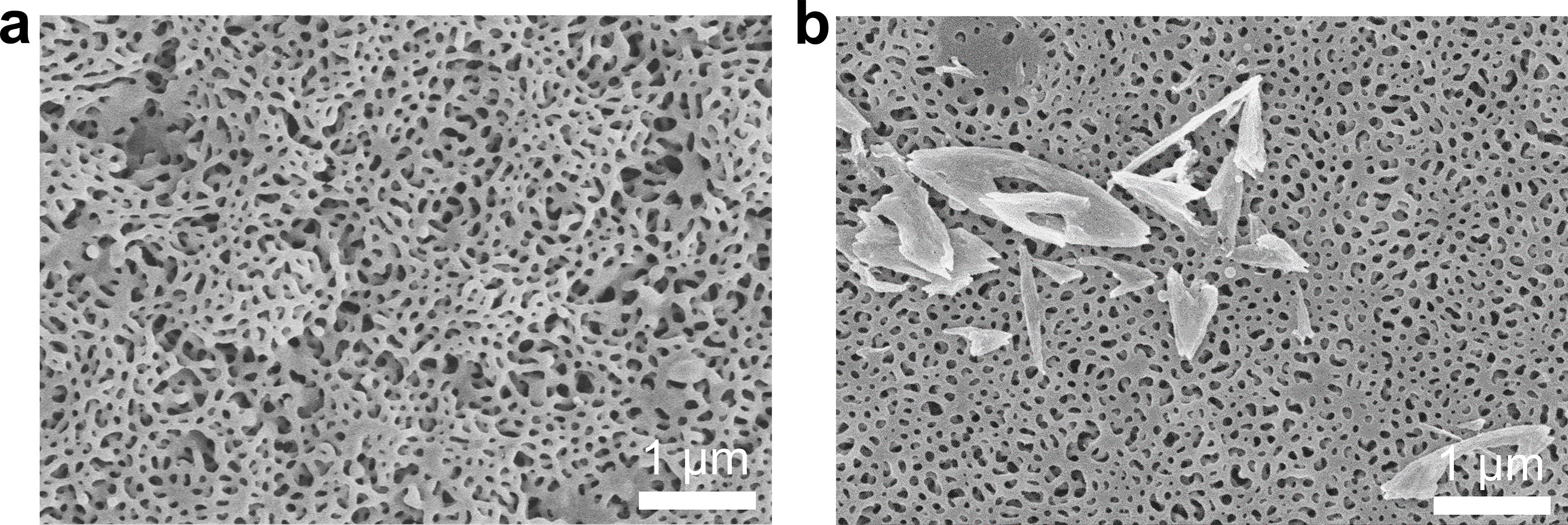
**

**Figure S1.** The SEM surface images of the BCP membranes before (a) and after soaking in the aqueous solution of ZIF-67 precursors for 1 h. The BCP membranes were produced by soaking the solid PS-*b*-P4VP films into toluene/methanol (V:V= 35/65) for 3 s followed by quenching in methanol and further air drying. 2D ZIF-67 seldom appeared on the BCP membranes, indicative of the inaccessible growth of ZIF-67 on the dried BCP membranes.

**
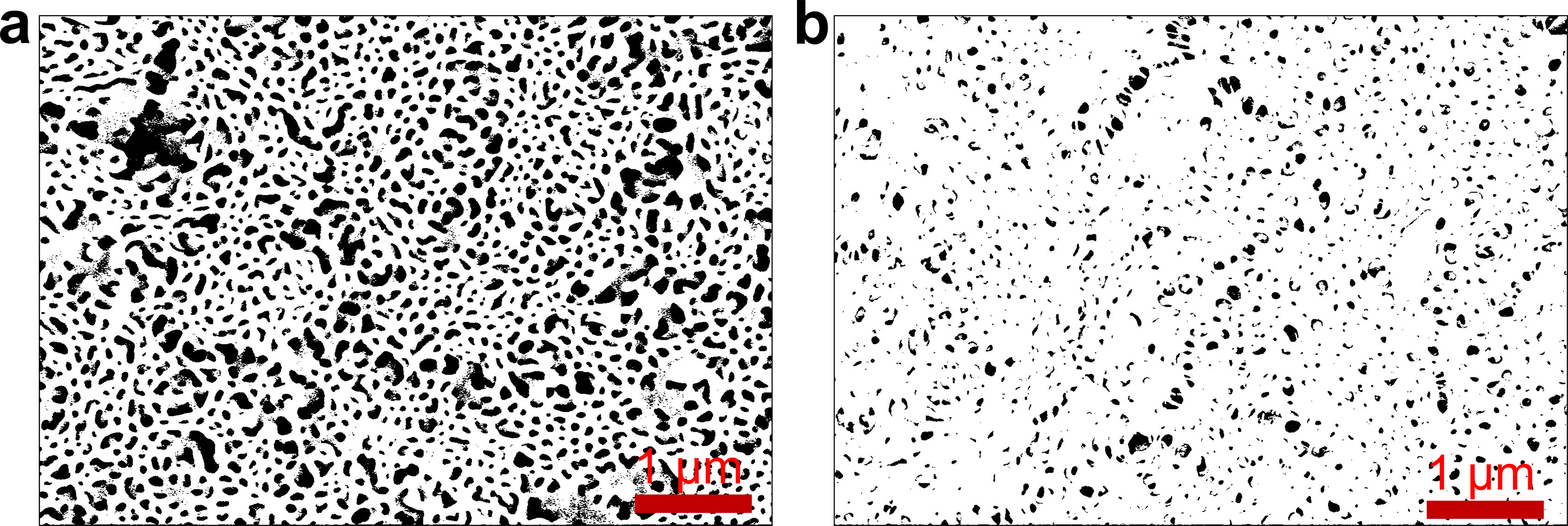
**

**Figure S2.** The corresponding binary images of Figure S1a (a) and Figure 1b (b) developed by *Image J*.

**
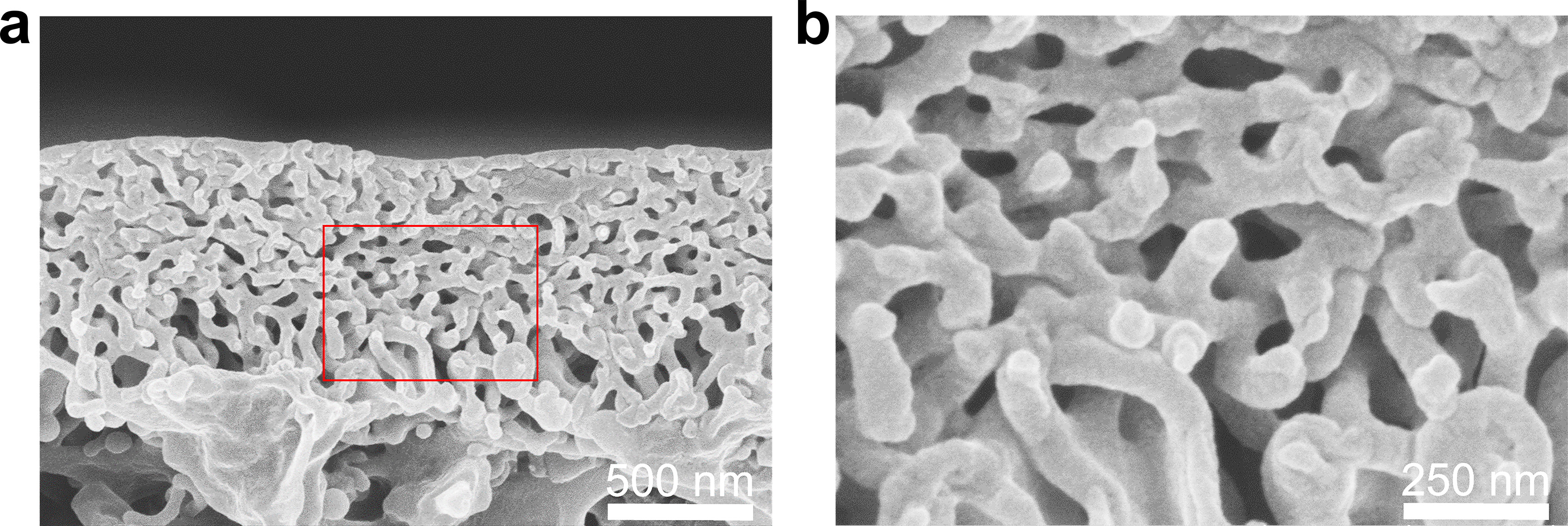
**

**Figure S3.** The SEM cross sections of the nanoporous BCP membranes produced by soaking the solid PS-*b*-P4VP films into toluene/methanol (V:V= 35/65) for 3 s followed by quenching in methanol and further air drying. (b) is the enlarged view of the red box shown in (a).

**
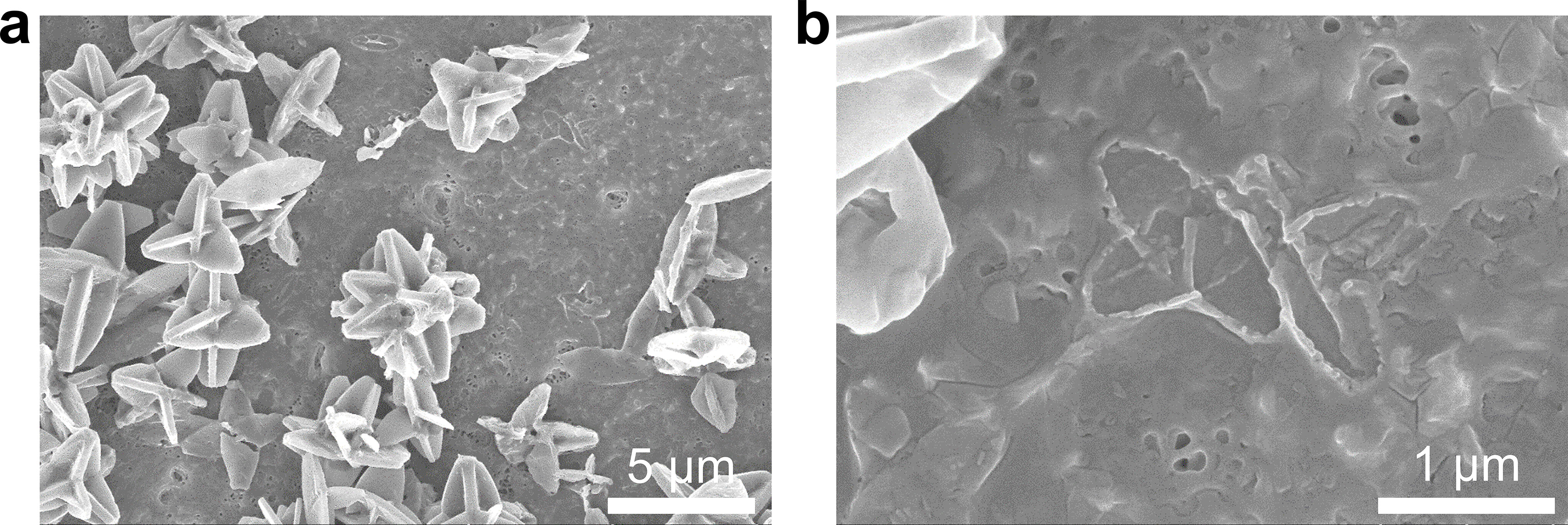
**

**Figure S4**. The SEM surface images of the BCP/ZIF-67 membranes (a) and the corresponding enlarged view (b). The BCP membranes produced by soaking the solid PS-*b*-P4VP films into toluene/methanol (V:V= 35/65) for 3 s followed by quenching in methanol. Then such membranes were taken out from methanol and instantly immersed into the aqueous solution of ZIF-67 precursors for 12 h followed by air drying.


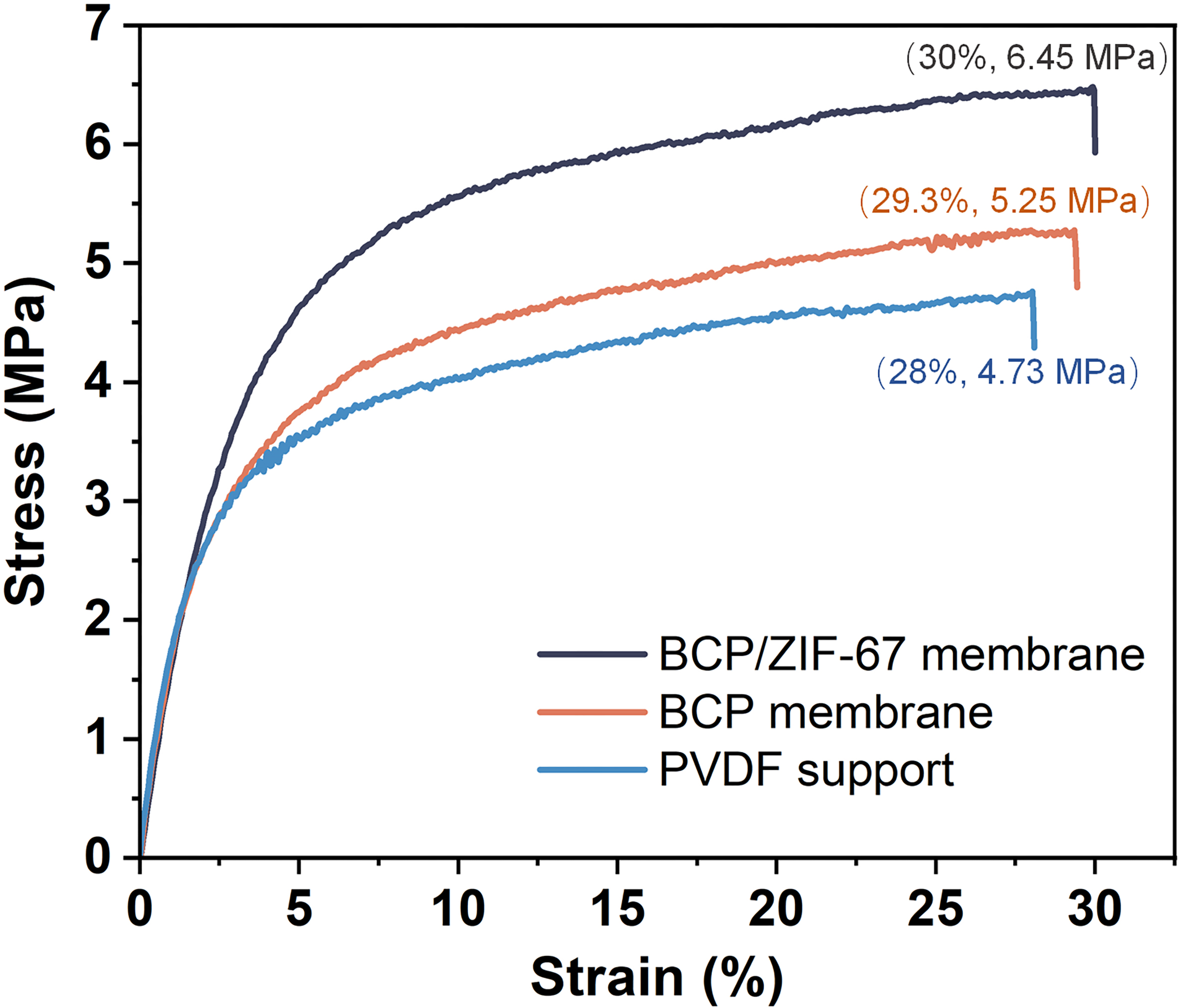


**Figure S5.** Stress-strain curves of the membranes with different compositions.


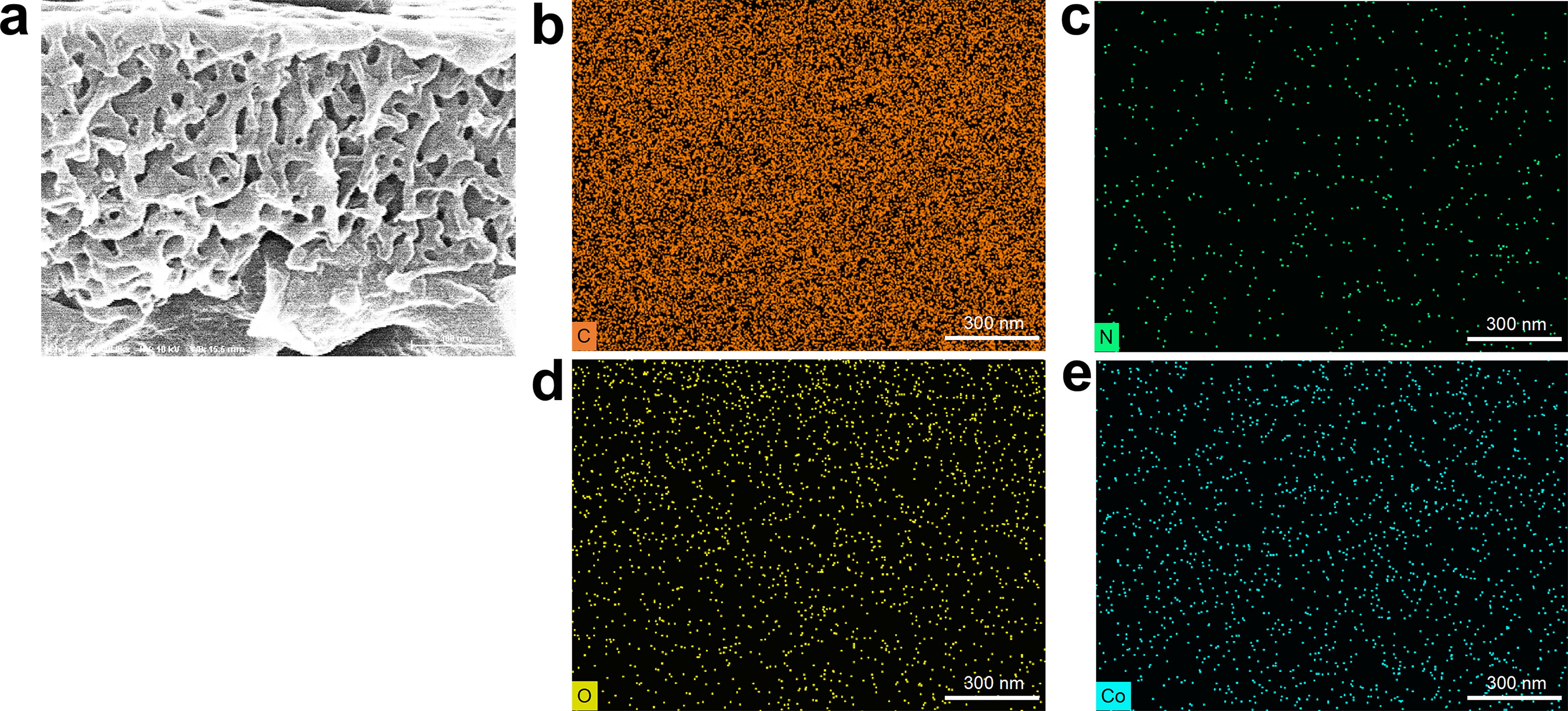


**Figure S6**. The EDS maps of the cross section of nanoporous BCP/ZIF-67 membrane soaked in mixed solvents containing toluene and methanol with the volume ratios of 35/65 for 3 s.

**
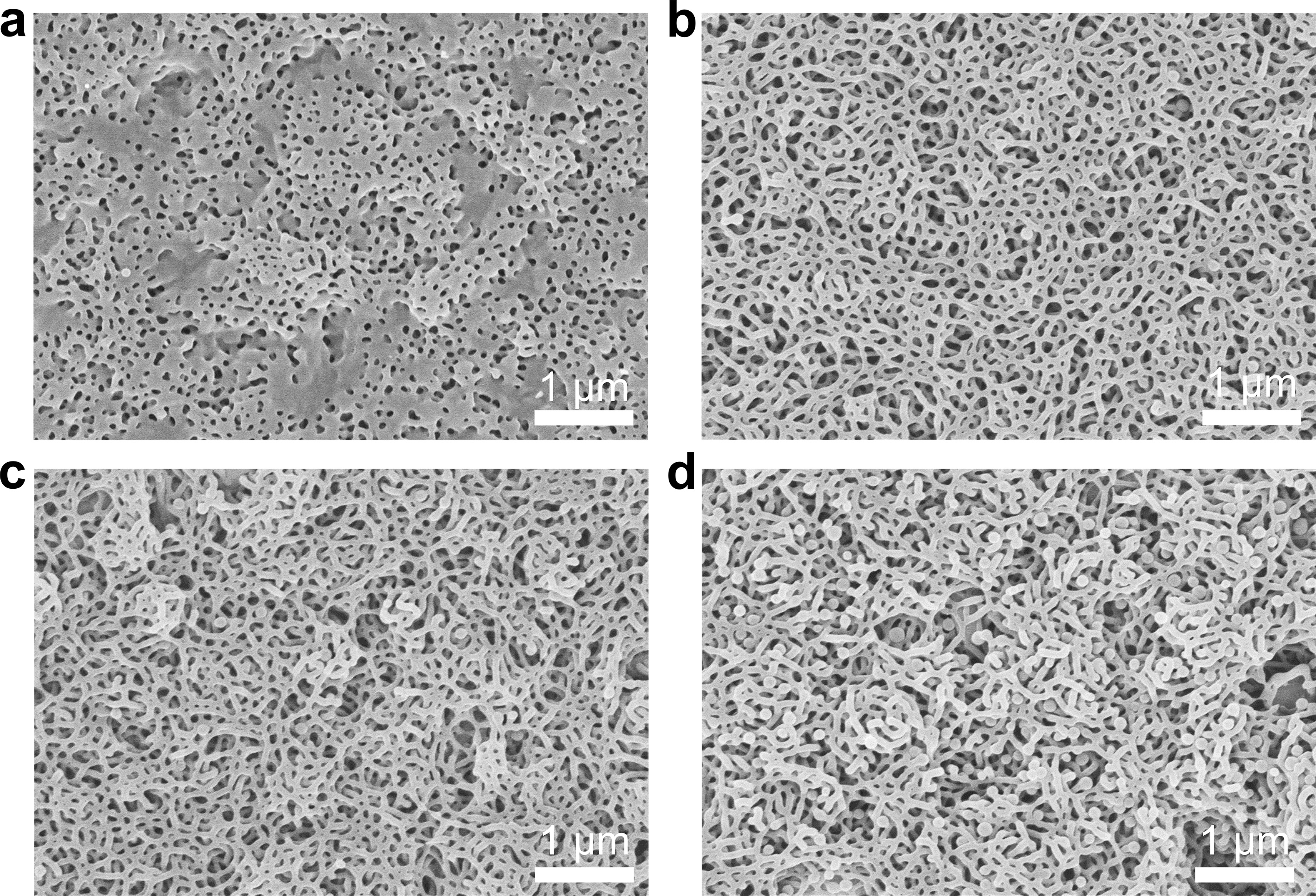
**

**Figure S7.** The SEM surface images of the BCP membranes prepared by soaking the solid BCP films into toluene/methanol with the volume ratios of 30/70 (a), 40/60 (b), 45/55 (c), and 50/50 (d) for 3 s, respectively, followed by quenching in methanol and further air drying.


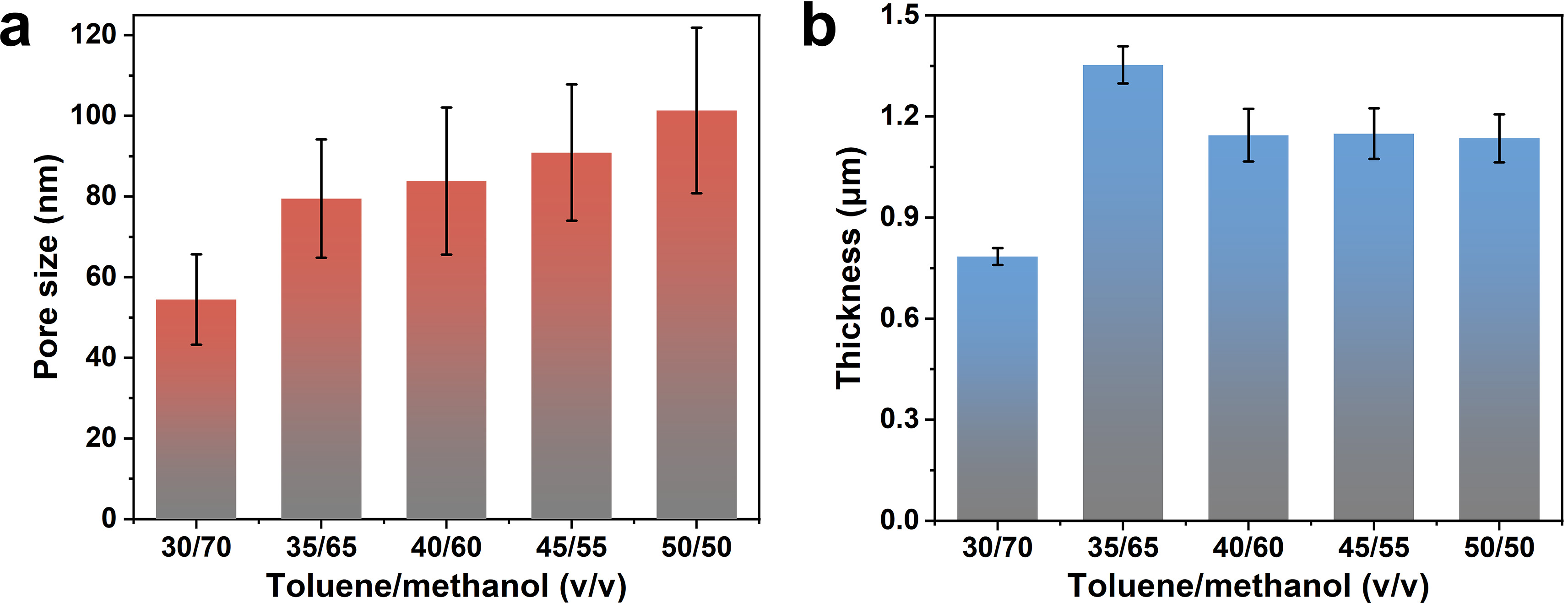


**Figure S8.** Histograms of pore size (a) and thickness (b) of the BCP membranes prepared by the “retarded dissolution” pore-forming strategy using the mixed solvents with different volume ratios of toluene to methanol for 3 s.


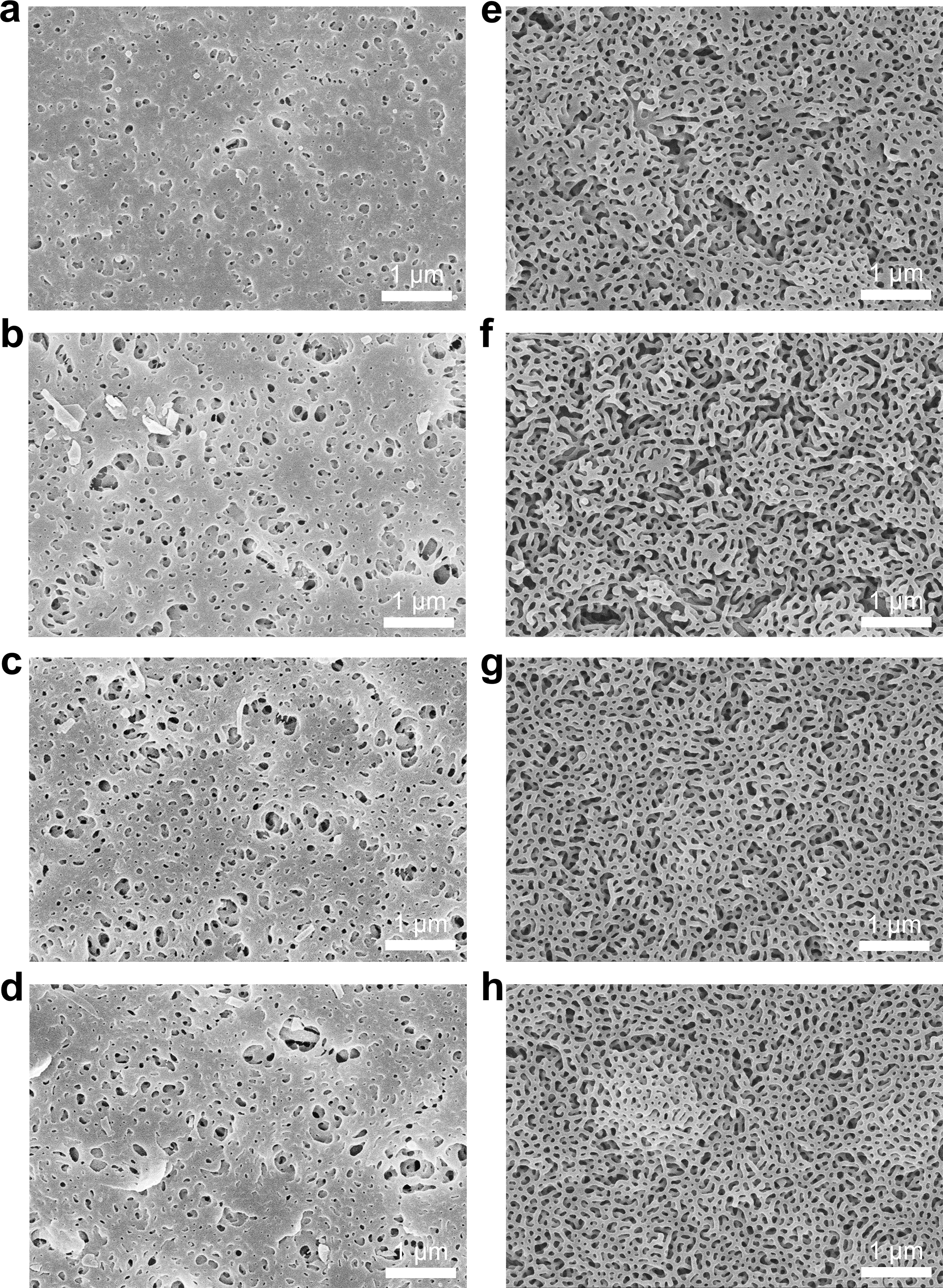


**Figure S9.** The SEM surface images of the BCP/ZIF-67 membranes (a-d) and the BCP membranes (e-h) soaked in the mixed solvents containing toluene and methanol with the volume ratios of 35/65 for 1 s (a, e), 5 s (b, f), 7 s (c, g), and 9 s (d, h), respectively.

**
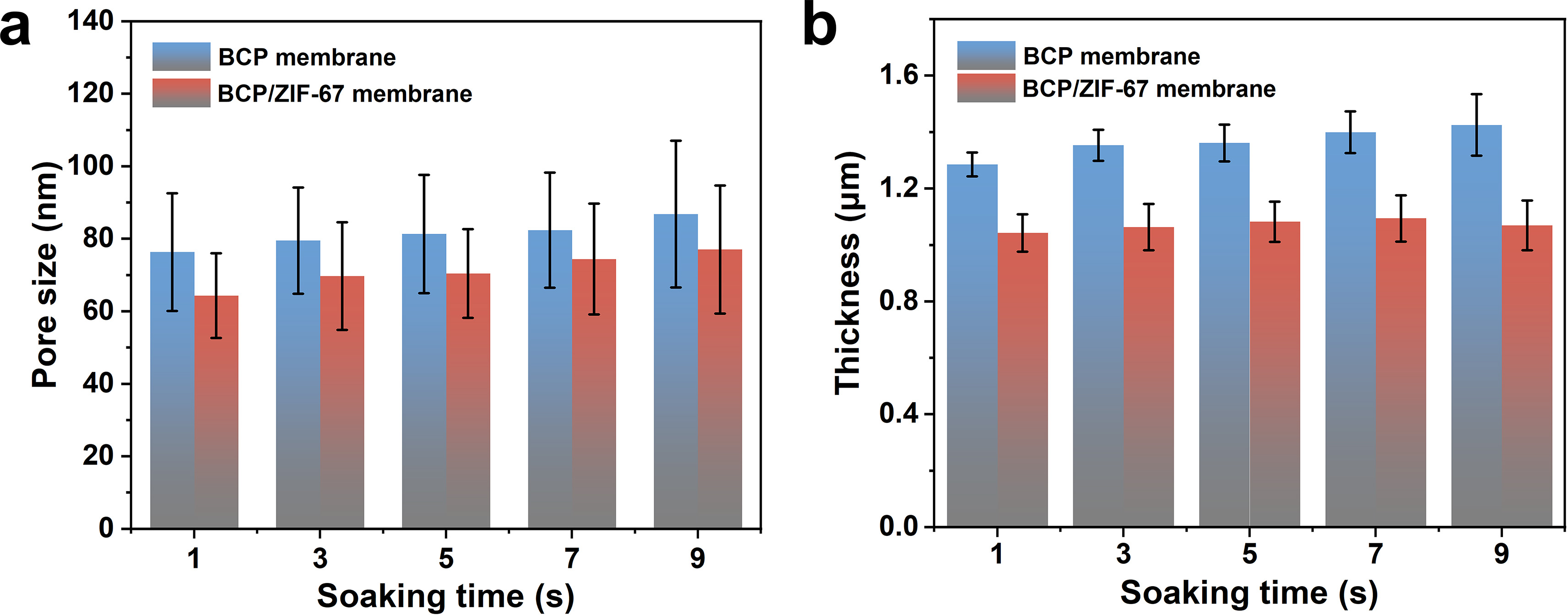
**

**Figure S10.** Histograms of pore size (a) and thickness (b) of the BCP/ZIF-67 membranes and the BCP membranes soaked in the mixed solvents containing toluene and methanol with the volume ratios of 35/65 for different durations.


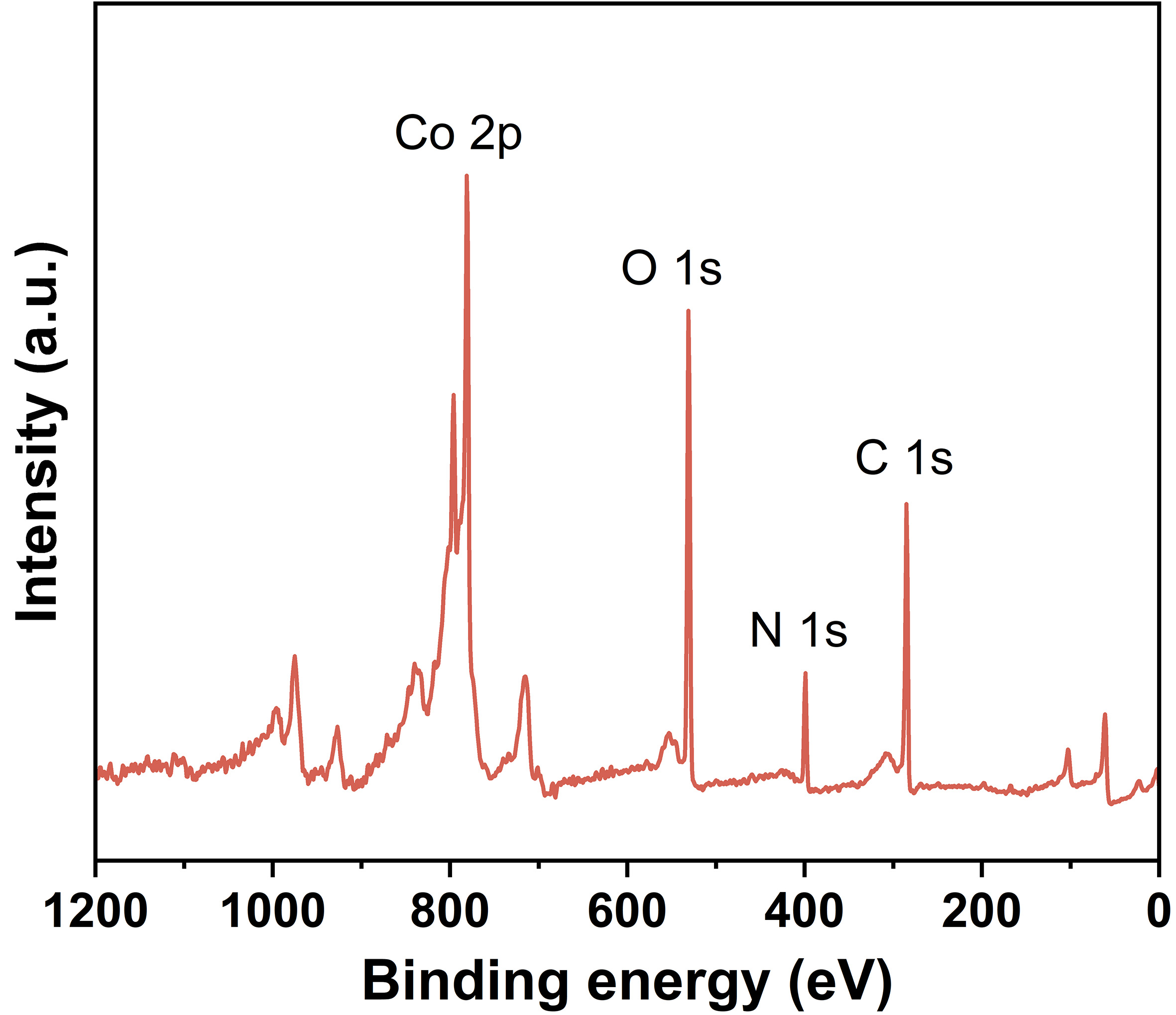


**Figure S11.** The full XPS scan of the BCP/ZIF-67 membrane after 2 hours of degradation experiment.


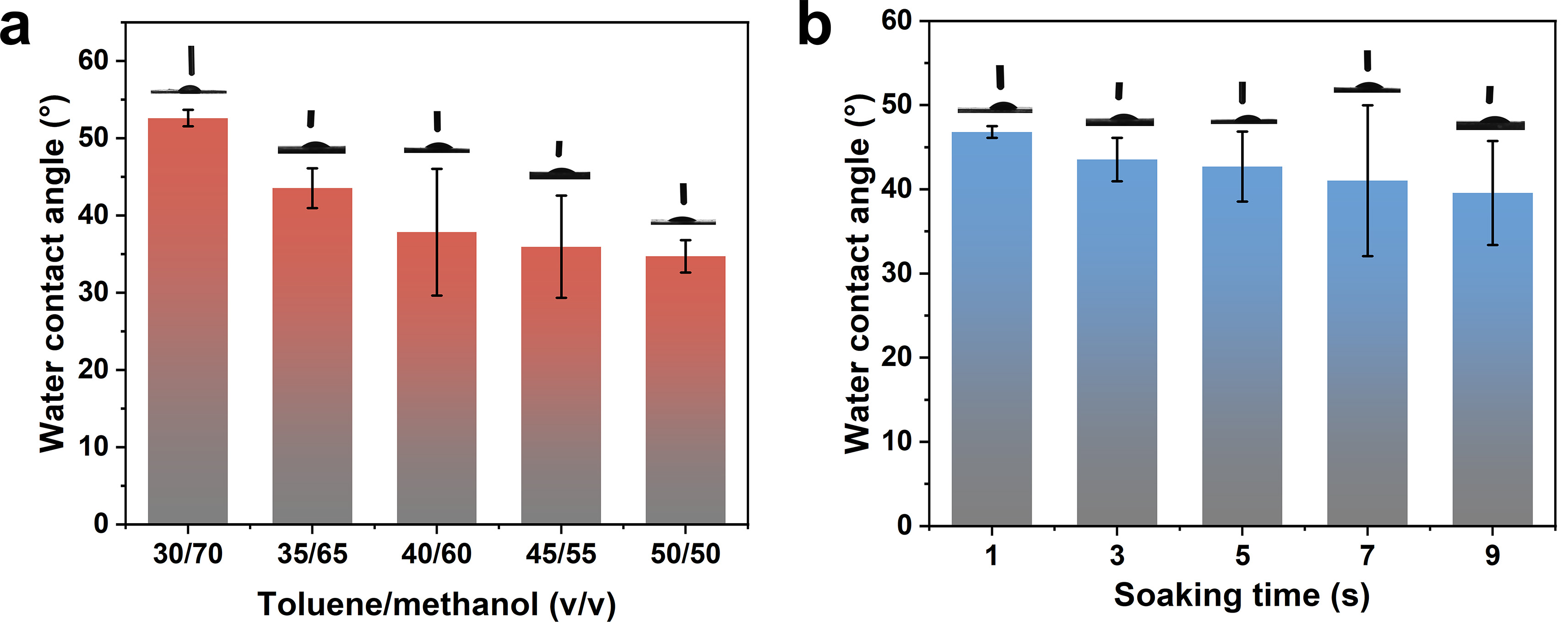


**Figure S12**. (a) The water contact angle of the BCP/ZIF-67 membranes prepared by soaking in the mixed solvents containing toluene and methanol with different volume ratios for 3 s. (b) The water contact angle of the BCP/ZIF-67 membranes prepared by soaking in the mixed solvents containing toluene and methanol with the volume ratio of 35/65 for various durations.


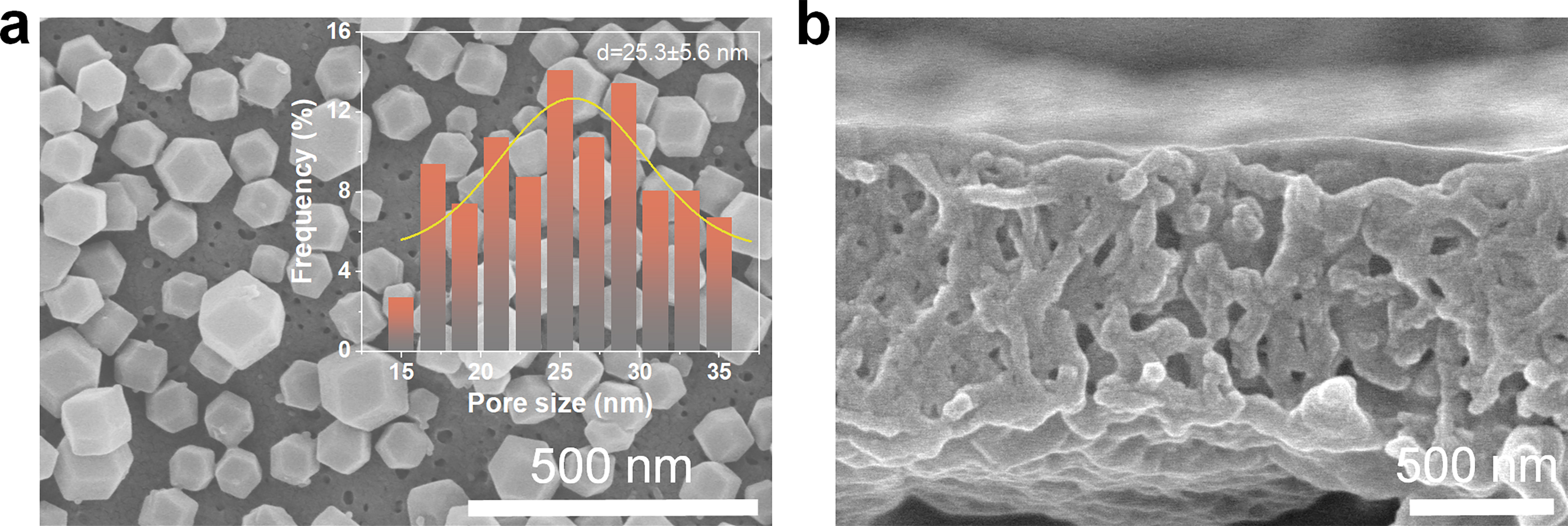


**Figure S13**. The SEM surface (a) and cross-sectional (b) images of the catalytic membrane constructed from PS-*b*-P4VP and 3D ZIF-67. The inset of (a) was the histogram of the pore size distribution of the prepared membrane.


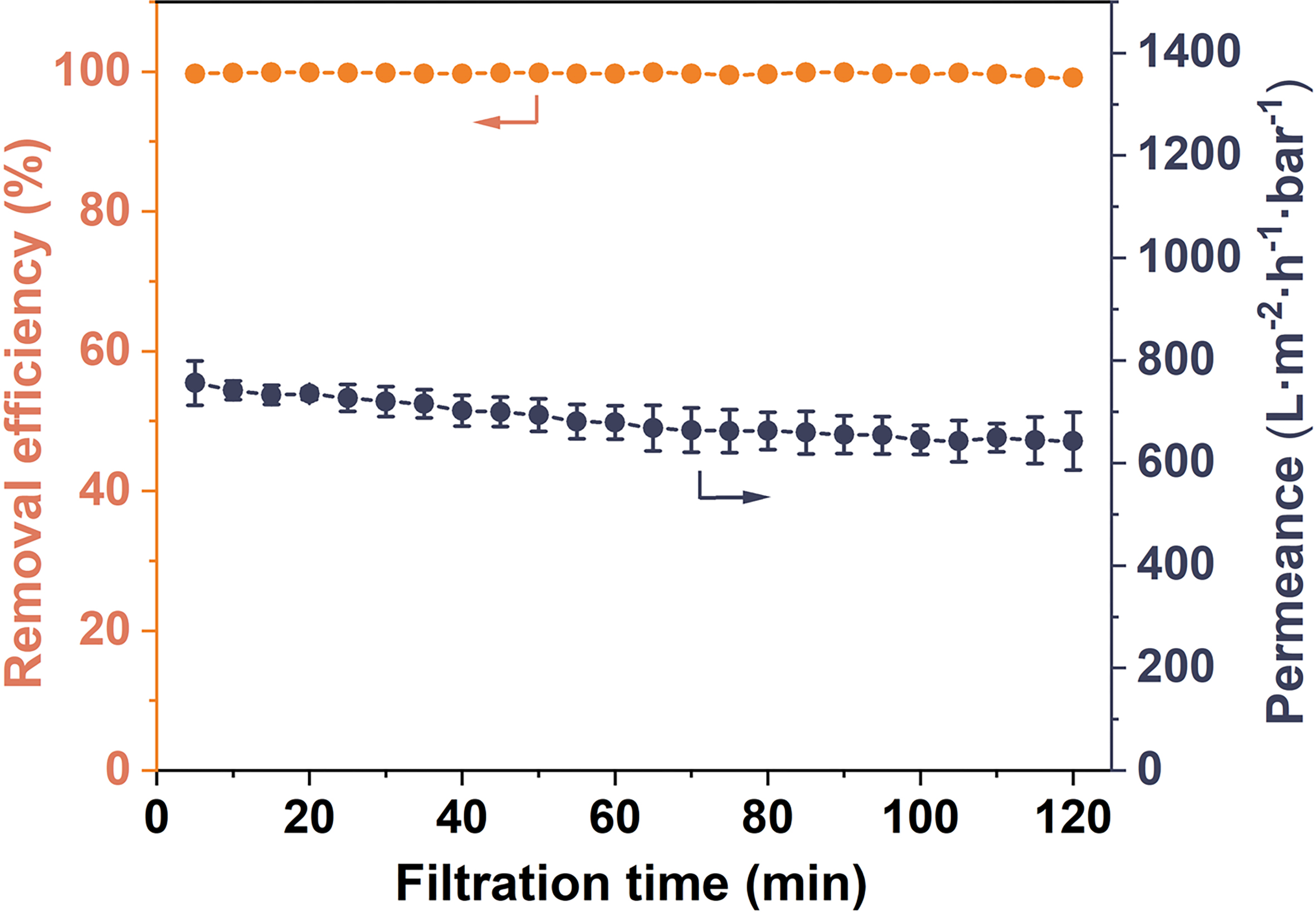


**Figure S14.** Continuous dynamic catalytic process of the membrane with PMS during 120 min of filtration. The catalytic membrane was constructed from PS-*b*-P4VP and 3D ZIF-67.


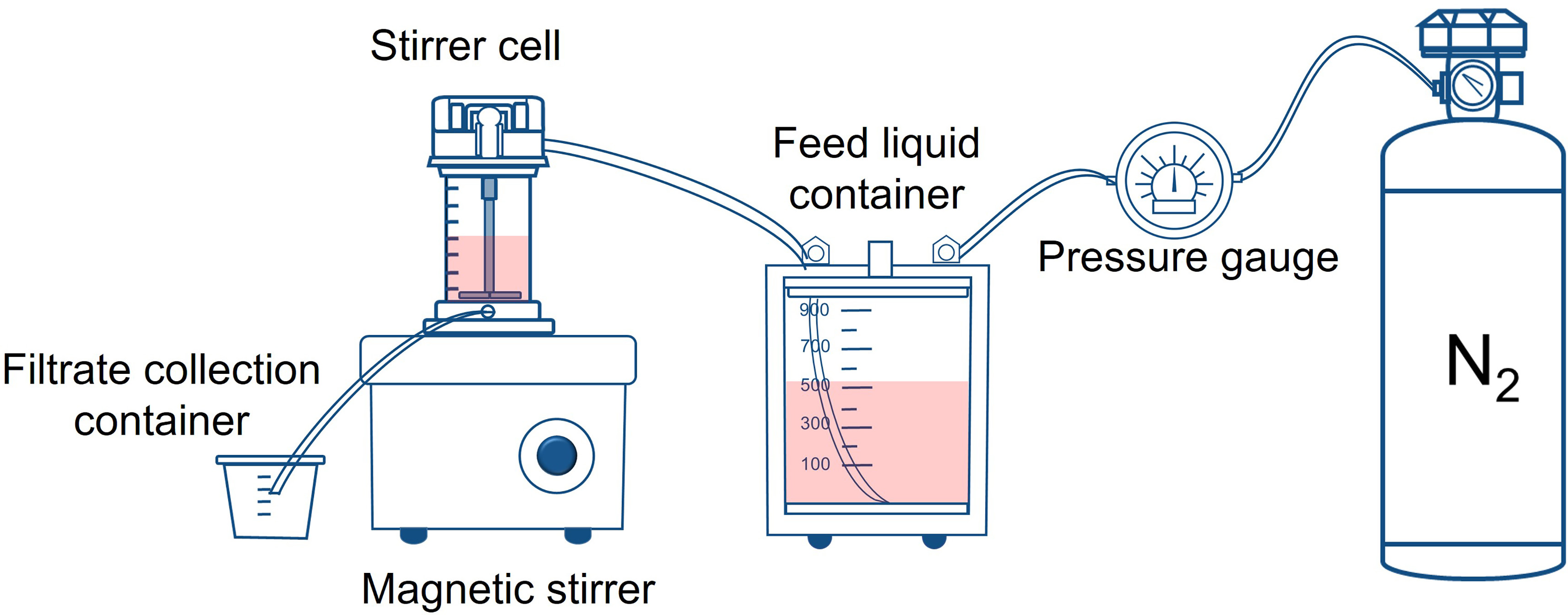


**Figure S15.** Schematic drawing of the homemade device to evaluate the catalytic capability of the synthesized hybrid membranes.

**Table S1.** Comparison of permeance and removal efficiency of the BCP/ZIF-67 membrane with the previously developed advanced membranes.

| **Material** | **Pollutant** | **PMS dosage (g/L)** | **Removal efficiency**  **(%)** | **Permeance**  **(L/(m^2^·h·bar))** | **Operation pressure (bar)** | **Flux (L/(m^2^·h))** | **Ref.** |
| --- | --- | --- | --- | --- | --- | --- | --- |
| 2D FeCoS@N-rGO | 30 mg/L MB^[a]^ | 0.2 | 92.4 | 235.5 | 1 | 235.5 | ^[12]^ |
| ZIF-67@PVDF | 20 mg/L AO7^[b]^ | 1 | 97.3 | 198.3 | 1 | 198.3 | ^[13]^ |
| Sm_0.5_Sr_0.5_CoO_3_-δ | 20 mg/L RhB^[c]^ | 0.307 | 100 | 666.7 | 0.9 | 600 | ^[14]^ |
| NiCo-LDH-GTP/GO | 10 mg/L MB^[a]^ | 0.768 | 98.9 | 196.5 | 1 | 196.5 | ^[15]^ |
| CuO modified membrane | 1 mg/L MB^[a]^ | 0.154 | 93.2 | 70 | 1 | 70 | ^[16]^ |
| Co@NCNT-MS/GO nanosheet membrane | 3 mg/L RhB^[c]^ | 0.501 | 100 | 70 | 1 | 70 | ^[17]^ |
| Fe-CCN nanosheet membrane | 10 mg/L BPA^[d]^ | 0.615 | 100 | 286 | 0.4 | 114.4 | ^[18]^ |
| OCN-Co/Fe/PVDF | 1 mg/L ATZ^[e]^ | 0.4 | 100 | 571 | 0.175 | 100 | ^[19]^ |
| MCO@PES | 10 mg/L TC^[f]^ | 0.246 | 99.1 | 153 | 0.4 | 61.2 | ^[20]^ |
| Co_SA_-PMOF | 4 mg/L MOX^[g]^ | 0.154 | 100 | 160 | 0.3 | 48 | ^[21]^ |
| ACO-O_V_@PES | 10 mg/L TC^[f]^ | 0.246 | 100 | 463 | 0.6 | 370.4 | ^[22]^ |
| Co-TiO_x_ | 5 mg/L RNTD^[h]^ | 0.05 | 100 | 131 | 1 | 131 | ^[23]^ |
| BCP/ZIF-67 | 10 mg/L RhB^[c]^ | 0.1 | 100 | 1866 | 0.1 | 186.6 | This  work |
|  | 10 mg/L RhB^[c]^ |  | 95 | 1838 |  | 183.8 |  |
|  | 10 mg/L RhB^[c]^ |  | 96.4 | 2117 |  | 211.7 |  |
|  | 10 mg/L RhB^[e]^ |  | 92.9 | 2593 |  | 259.3 |  |
|  | 10 mg/L RhB^[e]^ |  | 89.6 | 3070 |  | 307 |  |
|  | 10 mg/L RhB^[e]^ |  | 83 | 3800 |  | 380 |  |

^a^Methylene blue; ^b^Acid Orange 7; ^c^Rhodamine B; ^d^Bisphenol A; ^e^Atrazine; ^f^Tetracycline; ^g^Moxifloxacin; ^h^Ranitidine.

**Table S2.** Comparison of the long-term stability of the state-of-the-art catalytic membranes reported previously with the developed BCP/ZIF-67 hybrid membranes.

| **Membrane** | **Pollutant** | **Reaction time** | **Removal efficiency** | **Ref.** |
| --- | --- | --- | --- | --- |
| Fe SAC | 1 mg/L NPR^[a]^ | 10 h | 97.4% | ^[24]^ |
| OCN-Co/Fe/PVDF | 5 mg/L CIP^[b]^ | 24 h | 96% | ^[25]^ |
| Co-TiO_x_ | 5 mg/L RNTD^[c]^ | 100 h | 100% | ^[23]^ |
| CoAl-LDH | 1 mg/L RNTD^[c]^ | 29 h | 91% | ^[26]^ |
| Fe-CCN nanosheet membrane | 10 mg/L BPA^[d]^ | 15 h | 95% | ^[18]^ |
| CZO fibrous membrane | 100 mg/L RhB^[e]^ | 10 h | 95% | ^[27]^ |
| Sm_0.5_Sr_0.5_CoO_3-δ_ | 20 mg/L RhB^[e]^ | 100 h | 100% | ^[14]^ |
| CoAl-layered metal oxide membrane | 1 mg/L RNTD^[c]^ | 29h | 90% | ^[28]^ |
| LDH MOF | 10 mg/L RNTD^[c]^ | 28 h | 98% | ^[29]^ |
| BCP/ZIF-67 | 10 mg/L RhB^[e]^ | 68 h | 100% | This work |

^a^Nitenpyram; ^b^Ciprofloxacin; ^c^Ranitidine; ^d^Bisphenol A; ^e^Rhodamine B.

**References**

[S1] N. Li, G. Chen, J. Zhao, B. Yan, Z. Cheng, L. Meng, V. Chen, *J. Membr. Sci.* **2019**, *591*, 117341-117349.

[S2] G. Kresse, J. Furthmuller, *Phys. Rev. B* **1996**, *54*, 11169-11186.

[S3] S. Grimme, S. Ehrlich, L. Goerigk, *J. Comput. Chem.* **2011**, *32*, 1456-1465.

[S4] M. Ernzerhof, G. E. Scuseria, *J. Chem. Phys.* **1999**, *110*, 5029-5036.

[S5] C. Wang, Y. Wan, S. Yang, Y. Xie, S. Chu, Y. Chen, X. Yan, *Adv. Funct. Mater.*  **2024**, *34*, 2313596-2313605.

[S6] D.-H. Lim, A. S. Negreira, J. Wilcox, *J. Phys. Chem. C* **2011**, *115*, 8961-8970.

[S7] Z. Xu, G. Luo, Y. a. Fan, Y. Xia, J. Li, J. Zhang, K. Shih, Y. Tang, *Chem. Eng. J.* **2023**, *469*, 143867-143880.

[S8] M.-R. Li, G.-C. Wang, *J. Catal.* **2018**, *365*, 391-404.

[S9] K. Momma, F. Izumi, *J. Appl. Crystallogr.* **2011**, *44*, 1272-1276.

[S10] V. Wang, N. Xu, J.-C. Liu, G. Tang, W.-T. Geng, *Comput. Phys. Commun.* **2021**, *267*, 108033-108051.

[S11] Y. Wang, Z. Ao, H. Sun, X. Duan, S. Wang, *Appl. Catal. B: Environ.* **2016**, *198*, 295-302.

[S12] J. Ye, J. Dai, D. Yang, C. Li, Y. Yan, Y. Wang, *Chem. Eng. J.* **2021**, *418*, 129383-129396.

[S13] D. Liu, J. Yin, H. Tang, H. Wang, S. Liu, T. Huang, S. Fang, K. Zhu, Z. Xie, *Sep. Purif. Technol.* **2021**, *279*, 119755-119764.

[S14] J. Liang, K. Gao, A. Zhou, Y. Fang, S. Su, L. Fu, M. Sun, X. Duan, *Appl. Catal. B: Environ.* **2023**, *327*, 122440-122452.

[S15] R. Zhang, J. Zhao, J. Ye, X. Tian, L. Wang, J. Pan, J. Dai, *J. Hazard. Mater.* **2024**, *468*, 133793-133808.

[S16] S. Wang, J. Tian, Z. Wang, Q. Wang, J. Jia, X. Hao, S. Gao, F. Cui, *Chem. Eng. J.* **2020**, *396*, 125289-125299.

[S17] Y. Li, Y. He, J. Zhuang, H. Shi, *Chem. Eng. J.* **2022**, *430*, 133124-133136.

[S18] B. Chen, M. Zhang, L. Wang, L. Li, Q. Han, X. Liu, M. Wang, B. Liu, Y. Jiang, Z. Wang, *Appl. Catal. B* **2025**, *363*, 124827-124837.

[S19] P. Xu, R. Wei, P. Wang, T. Shen, T. Zheng, G. Zhang, *Environ. Sci. Technol.* **2024**, *58*, 17464-17474.

[S20] X. Zhang, S. Liu, Z. Wang, K. Feng, S. Xu, X. Li, P. Yu, X. Fan, H. Zheng, Y. Sun, *Chem. Eng. J.* **2023**, *477*, 146987-147000.

[S21] C. Yang, S. Shang, Y. Fan, K. Shih, X.-y. Li, L. Lin, *Appl. Catal. B* **2023**, *325*, 122344-122356.

[S22] X. Zhang, S. Xu, K. Feng, X. Li, P. Yu, Q. Liu, J. Zhang, X. Fan, C. Liu, H. Zheng, Y. Sun, *Water Res.* **2024**, *254*, 121351-121365.

[S23] C. Meng, B. Ding, S. Zhang, L. Cui, K. K. Ostrikov, Z. Huang, B. Yang, J.-H. Kim, Z. Zhang, *Nat. Commun.* **2022**, *13*, 4010-4019.

[S24] R. Guo, C. Guo, Z. Bi, H. Zhang, N. Lv, B. Xi, G. Hu, J. Xu, *Appl. Catal. B* **2024**, *356*, 124243-124252.

[S25] Z. Wang, Z. Yi, L. W. Wong, X. Tang, H. Wang, H. Wang, C. Zhou, Y. He, W. Xiong, G. Wang, G. Zeng, J. Zhao, P. Xu, *Adv. Mater.* **2024**, *36*, 2404278-2404290.

[S26] M. B. Asif, H. Kang, Z. Zhang, *J. Hazard. Mater.* **2022**, *425*, 127988-127990.

[S27] X. Zhang, J. Liu, H. Zhang, Z. Wan, J. Li, *Appl. Catal. B: Environ.* **2023**, *327*, 122429-122438.

[S28] M. B. Asif, H. Kang, Z. Zhang, *Chem. Eng. J.* **2023**, *463*, 142340-142353.

[S29] A. Sharmin, G. Zhang, M. A. Bhuiyan, B. Kumar Pramanik, *Chem. Eng. J.* **2024**, *495*, 153572-153585.
